# Geometry-induced spin chirality in a non-chiral ferromagnet at zero field

**DOI:** 10.1038/s41565-025-02055-3

**Published:** 2025-12-04

**Authors:** Mingran Xu, Axel J. M. Deenen, Huixin Guo, Pamela Morales-Fernández, Sebastian Wintz, Elina Zhakina, Markus Weigand, Claire Donnelly, Dirk Grundler

**Affiliations:** 1 Institute of Materials (IMX), School of Engineering, École Polytechnique Fédérale de Lausanne (EPFL), Lausanne, Switzerland; 2 Max Planck Institute for Chemical Physics of Solids, Dresden, Germany; 3 Institute of Applied Physics, TU Wien, Vienna, Austria; 4 Helmholtz-Zentrum Berlin für Materialien und Energie GmbH, Berlin, Germany; 5 International Institute for Sustainability with Knotted Chiral Meta Matter (WPI-SKCM2), Hiroshima University, Hiroshima, Japan; 6 Institute of Electrical and Micro Engineering (IEM), School of Engineering, École Polytechnique Fédérale de Lausanne (EPFL), Lausanne, Switzerland

**Keywords:** Magnetic properties and materials, Spintronics, Magnetic devices

## Abstract

Spin chirality is a fundamental property that manifests non-reciprocal transport—magnetochiral anisotropy (MChA). However, the application of MChA in technology is constrained by the necessity for an external magnetic field, complex non-centrosymmetric crystal synthesis and cryogenic environments. Here we overcome these challenges by imprinting geometric chirality onto a nickel tube via three-dimensional nanoengineering. We use two-photon lithography to create a structurally twisted polymeric template with micrometre-sized pitch and diameters and cover it with a uniform 30-nm-thick nickel shell. The nickel tube exhibits spontaneous MChA—non-reciprocal transport at zero magnetic field and room temperature. X-ray magnetic circular dichroism microscopy confirms helical spin textures stabilized by the torsion- and curvature-engineered shape anisotropy, while inelastic light scattering spectroscopy demonstrates robust non-reciprocal magnon transport at remanence, reconfigurable via magnetic field history. The chiral parameter in our device surpasses that of natural chiral magnets such as Cu_2_OSeO_3_. Analytical theory and micromagnetic simulations demonstrate that the non-reciprocity is further enhanced by downscaling the feature sizes. Our results establish a scalable, geometry-driven nanotechnology that imprints spin chirality on non-chiral ferromagnets and may enable nanoscale integration of chirality-enhanced magnonics and spintronics for real-world use cases.

## Abstract (web summary)

Three-dimensional chiral magnetic tubes created via two-photon lithography and nickel film deposition exhibit chiral spin textures and non-reciprocal magnon transport.

## Main

Spin chirality arises from broken inversion symmetry in crystalline structures and gives rise to anisotropic transport under a magnetic field, a phenomenon known as magnetochiral anisotropy (MChA)^1,2^. This transport anisotropy contrasts with magnetic anisotropy, which arises from energy differences associated with variations in the direction of magnetization in the ground state of magnetically ordered materials such as ferromagnets. MChA has emerged as a key concept in quantum materials science, manifesting in diverse systems such as superconducting diode effects^3,4^, chiral-induced spin selectivity in molecular systems^5^ and chiral anomalies in Weyl semimetals^6,7^. Beyond fundamental research, MChA holds immense industrial potential, particularly in advancing electrical communication^8,9^ and energy-efficient logic/memory applications^10–13^.

Fundamentally, MChA arises from the simultaneous breaking of time-reversal symmetry (*T*) and spatial inversion symmetry (*P*), which lifts the degeneracy of the energy bands *ε*, and enforces the non-reciprocity: $\varepsilon \left({\mathbf{k}}\right)\ne \varepsilon \left(-{\mathbf{k}}\right)$, with **k** the wavevector^7^. In magnetic systems with magnetization *M*, the breaking of *PT* symmetry gives rise to a toroidal moment ${\mathbf{\uptau }}{\boldsymbol{\propto }}\iint {\rm{d}}{A}{\mathbf{r}}\times {\mathbf{M}}$ (ref. ^14^) with **r** the coordinate vector and the integration is over a cross-section of area *A*. The toroidal moment facilitates the analysis of the energy-band asymmetry, where information carriers with wavevector **k** fulfilling ${\mathbf{k}}\cdot{\mathbf{\uptau }}\ne 0$ exhibit non-reciprocal transport.

Despite its importance, the industrial exploitation of MChA and miniaturization of corresponding devices remain constrained by intrinsic limitations. The energy associated with *PT* symmetry breaking is typically ∼1 meV. Electrical MChA is generally weak due to a mismatch in energy scales (∼1 eV for electrons), resulting in a small non-reciprocity factor of the order of ∼10^−3^. In contrast, magnons, low-energy excitations of magnetic order, naturally exist at much lower energy scales (∼10 μeV in ferromagnets) than electrons, making the non-reciprocity more pronounced. Natural chiral magnets inherently break *P* symmetry and host magnons^15,16^, but lack canted moments in their ground state^17^, necessitating an external magnetic field to break *T* symmetry^14^, which limits their scalability and practical device integration.

To overcome these limitations, new strategies for breaking *PT* symmetry are needed. Curved geometries inherently break *P* symmetry in magnetic systems^18–22^, leading to a non-local chiral symmetry-breaking effect^23–25^. This chiral symmetry breaking has been predicted to give rise to non-reciprocal magnon transport in ferromagnetic tubes^26–28^. Major challenges in realizing a sizeable effect include the need for high-precision three-dimensional (3D) nanoengineering technology compatible with ferromagnetic materials and detection methods capable of disentangling +**k** and −**k** states. A device to realize MChA in such systems has remained elusive^29–32^.

**Fig. 1:**
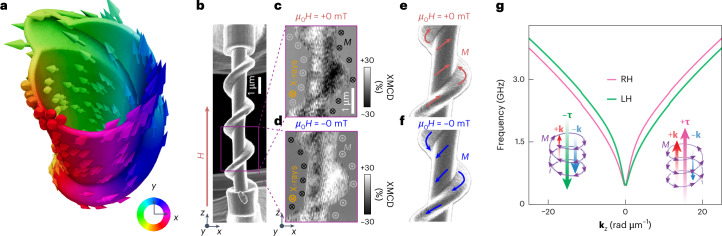
Screw-like spin textures of ACMs and asymmetric dispersion of magnons at zero magnetic field. **a**, Simulated spin texture of a right-handed ACM in the remanent state consisting of a hollow ferromagnetic tube with a surface spiral. The colour wheel represents the magnetization direction which inherits the screw-like topology. **b**, SEM image of a RH ACM, fabricated by conformal coating of a ferromagnetic nickel layer on a 3D scaffold. **c**,**d**, XMCD images measured at the nickel L_3_ absorption pre-edge for the segment marked with a purple square in **b**. The extracted spin configurations after application of +250 mT and −250 mT along the central axis of the ACMs are labelled by +0 mT (**c**) and −0 mT (**d**), and are sketched in **e** and **f**, respectively. **g**, Dispersion relation of the lowest magnon band on tubes with LH (green curve) and RH (purple) helical textures under a tilting angle of 20° with the longitudinal axis. Insets: spin configurations (purple arrows) and corresponding toroidal moment vectors **τ** motivating the non-reciprocal propagation of magnons (indicated via differently large wavevectors +**k** and −**k** collinear with **τ** and the *z* direction).

In this work, we introduce a scalable approach and realize spontaneous MChA by introducing a structural twist in a tubular nickel cylinder (Fig. 1). By engineering torsion- and curvature-induced shape anisotropy, we stabilize a spiralling spin texture with a defined handedness, visualized by X-ray magnetic circular dichroism (XMCD) microscopy. The spiralling spin state introduces a toroidal moment, resulting in magnon band asymmetry in polycrystalline nickel, and hence MChA. We term our samples artificial chiral magnets (ACMs). Using Brillouin light scattering, we directly measure -magnon propagation at remanence, which is controlled by the magnetic field history. Our results demonstrate how non-reciprocity is governed by the structural handedness of the geometric screw and the field history used to stabilize the spin chirality. Micromagnetic simulations and analytical theory reveal that the non-reciprocity arises from a combination of local and non-local interactions related to the curvature and topology of the nanostructure.

## Spontaneous MChA

The ACMs considered in this work are twisted hollow magnetic cylinders with an either right-handed (RH) or left-handed (LH) screw-like surface topology. They consist of a polymeric template created via two-photon lithography (TPL) covered by a uniform 30-nm-thick nickel shell deposited via atomic layer deposition (ALD). We refer to the straight tubular part of the ACM as the tube region and the helical surface topography as the helix region. In micromagnetic simulations we find that the presence of the spiralling screw imprints a spiralling spin texture at zero field in both the tube and the helix region (Fig. 1a). A scanning electron microscopy (SEM) image of a RH ACM is shown in Fig. 1b.

**Extended Data Fig. 1:**
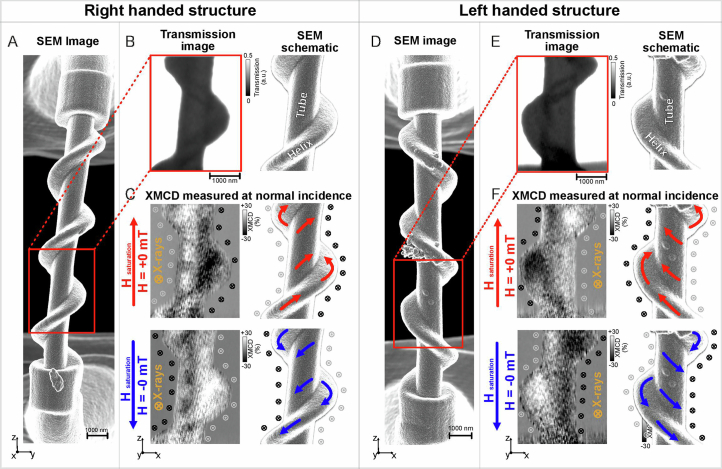
Screw-like spin texture of a right- and left-handed Artificial Chiral Magnets revealed by Scanning Transmission X-ray Microscopy. (**A**) Scanning electron microscope (SEM) image of a right-handed (RH) artificial chiral magnet (ACM). (**B**) Transmission image from the region highlighted in red in (A), acquired near the Ni L₃ absorption edge, alongside schematic of the ACM illustrating its tubular and helical components. (**C**) XMCD images in remanence ( ± 0 mT) after a saturating field ( ± 250 mT) along the ACM’s main axis, with interpretations overlaid on the SEM schematic, where ⊗ and ⊙ represent into-the-plane and out-of-the-plane magnetization directions, respectively. (**D**) SEM image of a left-handed (LH) artificial chiral magnet (ACM). (**E**) Transmission image from the region highlighted in red in (A), acquired at the Ni L₃ absorption off-edge, alongside schematic of the ACM illustrating its tubular and helical components. (**F**) X-ray magnetic circular dichroism (XMCD) images captured in remanence ( ± 0 mT) following the application of a saturating magnetic field ( ± 250 mT) along the main axis of the ACM. Interpretations of the experimental results are superimposed on the SEM schematic.

To determine the directionality of the magnetic configuration within the ACM samples, we performed scanning transmission X-ray microscopy (STXM) imaging (Methods). The magnetic configurations were measured at remanence (*µ*_0_*H* = 0 mT), after application of a large positive (+) or negative (−) magnetic field of *µ*_0_*H* = ±250 mT along the long axis ($\hat{z}$) of the structures. The resulting contrast in XMCD images measured at normal incidence revealed that the out-of-plane magnetic component was azimuthally oriented. As shown in Fig. 1c–f, the contrast in images labelled by +0 mT and −0 mT reversed sign with the opposing directions of the saturating field. For RH and LH ACMs (Extended Data Fig. 1), we observed textures with opposite helicity after application of +250 mT, confirming that the azimuthal component’s directionality was determined by the helical gyration direction of the structural screw. The spin chirality followed the corresponding structural chirality, generating oppositely oriented toroidal moments ∓**τ** and inverted magnon non-reciprocities **τ** · **k** (Fig. 1g), where **k** is the magnon wavevector, for LH and RH ACMs, respectively.

## Reprogrammable zero-field chiral magnon transport

**Fig. 2:**
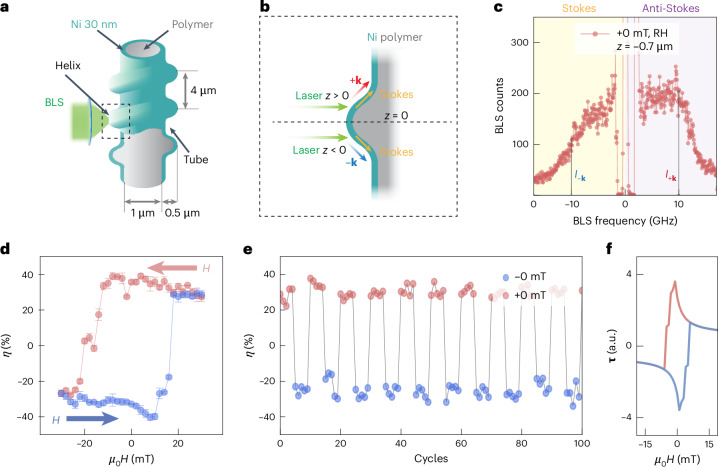
Reprogrammable non-reciprocal magnons of an ACM in the remanent state. **a**, Sketch of μBLS spectroscopy performed on an ACM. **b**, The different light scattering geometries at positions *z* < 0 µm and *z* > 0 µm lead to differently transferred magnon wavevectors as sketched for the Stokes signal. **c**, Magnon spectra obtained on a RH ACM in the remanent state +0 mT (after applying +30 mT) at position *z* = −0.7 µm. **d**, Intensity non-reciprocity *η* at 10 GHz evaluated at position *z* = −0.7 µm as a function of magnetic field and sweep direction (indicated by red and blue arrows) shows a hysteretic behaviour. Error bars represent the s.d. obtained from averaging over three consecutive measurements at each field point. **e**, Non-reciprocity parameter *η* extracted from spectra taken at *µ*_0_*H* = ±0 mT after applying a magnetic field of ±30 mT along axial directions of a RH ACM. *η* was measured at *f* = 10 GHz five consecutive times with the same polarity (cycles) at position *z* = −0.7 µm before reversing the magnetic field history. The data reflect the robust programmability of signal asymmetry (amplitude non-reciprocity) at zero field. **f**, Numerically computed toroidal moment **τ** of a RH ACM simulated as a function of field *H* and its sweep direction. The field was applied along the *z* direction and swept from negative to positive (blue lines) and back (red lines). The hysteretic behaviour leads to a reprogrammable **τ** of opposite sign at *µ*_0_*H* = 0 mT.

**Extended Data Fig. 2:**
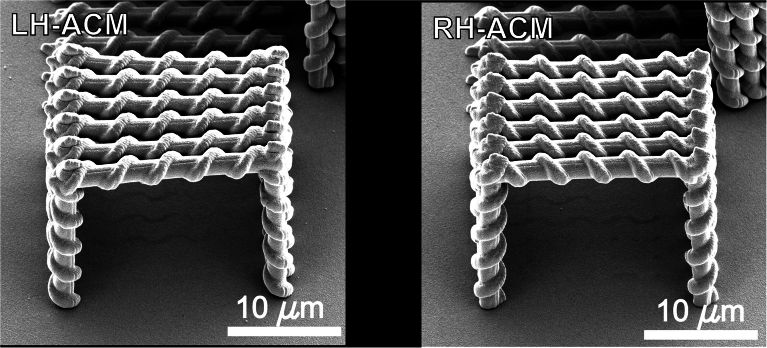
Scanning electron micrographs of arrays of left-handed (LH) and right-handed (RH) artificial chiral magnets (ACMs). The SEM images were acquired at a 30° tilt with respect to the substrate normal. Each array unit comprises a suspended ACM supported by two vertical chiral tubes, enabling top-side optical access. The LH-ACM and RH-ACM arrays were fabricated on the same substrate and received identical Ni coatings in the same ALD run. Geometric parameters (pitch, height, and tube diameter) are nominally the same; only the handedness differs.

The magnon dynamics and non-reciprocities are probed locally by means of microfocused Brillouin light scattering microscopy (μBLS) on thermally excited magnons at room temperature (Fig. 2a). An SEM image of a sample analysed using BLS is shown in Extended Data Fig. 2. The scattering process between photons and magnons in an opaque thin film obeys the momentum conservation law for the in-plane momenta (wavevectors). In the BLS spectrum, the Stokes (anti-Stokes) peak corresponds to the creation (annihilation) of magnons. In the μBLS set-up, the focused laser light offers a cone of incidence angles that is symmetric around the optical axis of the lens. If the laser light is focused on a curved surface whose normal direction does not coincide with the optical axis of the lens, the incidence-angle distribution becomes unbalanced with respect to +**k** or –**k** wavevectors. This imbalance follows the same principle as standard wavevector-resolved BLS spectroscopy^33,34^. It is illustrated in Fig. 2b, where depending on the laser spot position, the Stokes (anti-Stokes) signal is mainly due to either +**k** or –**k** (–**k** or +**k**) magnons. In the case of an asymmetric dispersion relation, a frequency asymmetry between Stokes and anti-Stokes peaks is resolved.

By analysing the Stokes and anti-Stokes signals of the thermally excited magnons, we identify differences in the resonance frequencies Δ*f* and the intensities *I*_±**k**_ of the BLS peaks (Fig. 2c). To demonstrate the control of the zero-field intensity non-reciprocity by magnetic field history, we define the asymmetry parameter $\eta = \frac{{I}_{+{\mathbf{k}}}-{I}_{-{\mathbf{k}}}}{\left({I}_{+{\mathbf{k}}}+{I}_{-{\mathbf{k}}}\right)}100 \%$, and analyse the intensities *I*_+**k**_ and *I*_−**k**_ in the Stokes and anti-Stokes spectrum, respectively, at the same characteristic frequency *f*. Here, *η* = 0% corresponds to a fully symmetric Stokes and anti-Stokes signal, and $|\eta| =100 \%$ to a maximally asymmetric signal.

In Fig. 2d,e, we present the extracted asymmetries. For BLS spectra measured at the same position on an ACM the opposing intensity asymmetries are attributed to opposing spin helix configurations considering the XMCD data of Fig. 1c,d. Figure 2c shows zero-field Stokes and anti-Stokes spectra with strikingly different signal intensities for negative and positive frequencies at *z* = −0.7 µm, respectively. Notably, this intensity contrast is reversed when the magnon spectrum is measured at *z* > 0, as shown for *z* = +0.9 µm in Supplementary Fig. 18. Opposite *z*-dependent spectral weights are observed in LH ACM tubes (Supplementary Fig. 16), reflecting the chirality-dependent nature of the magnon transport. Evaluated at a common frequency of *f* = 10 GHz at position *z* = −0.7 µm (indicated by vertical lines in Fig. 2c), the asymmetry is quantified by *η* = 35.7%, at zero field. Note that *z* = −0.7 µm and *f* = 10 GHz represents the optimized condition for probing *η* as shown in Supplementary Fig. 14, where the laser spot is partially positioned on both helix and tube region. Further BLS spectra were taken and evaluated, while an applied field was varied from +30 mT to −30 mT and then back to +30 mT in small field steps along the long axis. In Fig. 2d we display the intensity asymmetry parameters *η* evaluated at ±10 GHz (Supplementary Text). The asymmetry parameter *η* clearly follows a hysteresis curve with non-zero values over broad field regimes. We explored the programmability of this chiral memory effect by sequentially applying a magnetic field of +30 (−30) mT and extracting *η* for five consecutive measurements at +0 (−0) mT before reversing the magnetic field history. The results, presented in Fig. 2e, reveal that within the noise level, *η* exhibits only two levels. We attribute this behaviour to two different magnon reciprocities reproducibly induced at zero field.

We relate the reprogrammable asymmetries to the switching of the sense of handedness of the spin texture in the ACM (Fig. 1c,d), and the concomitant changing of the sign of the toroidal moment. The sign change of the toroidal moment is consistent with simulations performed on a miniaturized ACM (Fig. 2f and Supplementary Text).

## Quantifying the MChA

**Fig. 3:**
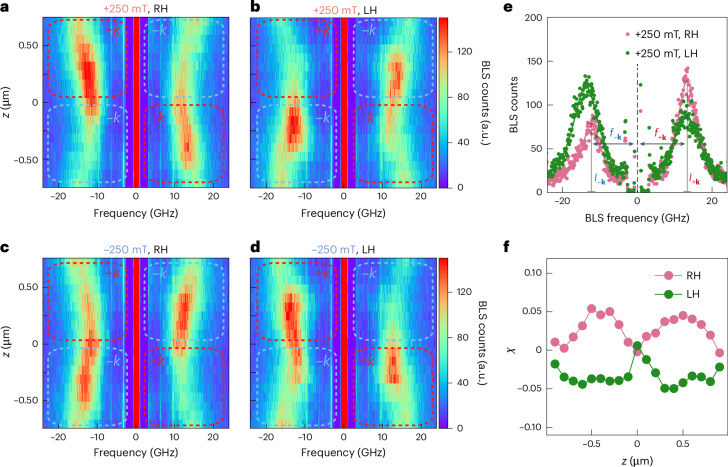
Asymmetry parameter *χ* of ACMs extracted from BLS spectra taken at ± 250 mT. **a**–**d**, BLS spectra measured as a function of position across the spiral (see definition of *z* in Fig. 2a) along a RH ACM in a field of +250 mT (**a**) and −250 mT (**c**), and along a LH ACM in a field of +250 mT (**b**) and −250 mT (**d**). The red/blue boxes indicated how we allocate signals to wavevectors of magnons, consistent with the colours in Fig. 2b. **e**, Magnon spectra obtained on a RH ACM (pink symbols) and a LH ACM (green symbols) in a field *µ*_0_*H* = +250 mT at position *z* = −0.5 µm. Curves reflect Lorentzian fits. **f**, Quantitative analysis of the frequency non-reciprocity parameter *χ* for a RH ACM (pink) and a LH ACM (green) as a function of position *z*.

In previous works on natural chiral magnets^35,36^, the strength of magnetochiral effects was quantified using *g*_MChA_ evaluated in a finite magnetic field. We use a nominally equivalent parameter $\chi =\frac{\Delta f{\prime} (+250\,\mathrm{mT})}{{f}_{+{\mathbf{k}}}\left(+250\,\mathrm{mT}\right)+{f}_{-{\mathbf{k}}}(+250\,\mathrm{mT})}-\frac{\Delta f{\prime} (-250\,\mathrm{mT})}{{f}_{+{\mathbf{k}}}\left(-250\,\mathrm{mT}\right)+{f}_{-{\mathbf{k}}}(-250\,\mathrm{mT})}$ with *f*_−**k**_ (*f*_+**k**_) the central peak frequency sampled at positive (negative) wavevectors (Fig. 3) and Δ*f*′ proportional to the difference in +**k** and −**k** peak frequencies to locally evaluate the relative magnitude of the frequency non-reciprocity at an external field of ±250 mT of both RH and LH ACMs (Supplementary Text).

**Extended Data Fig. 3:**
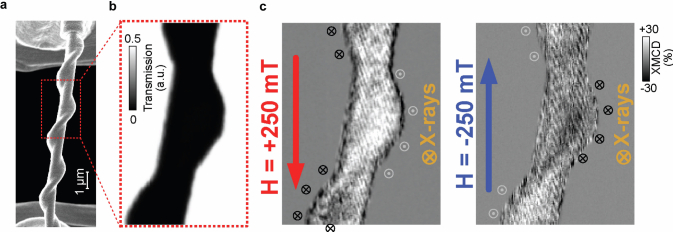
Scanning Transmission X-ray Microscopy on a right-handed Artificial Chiral Magnet at ± 250 mT. (**A**) SEM image of a right-handed (RH) artificial chiral magnet (ACM) with tube diameter≈500 nm. (**B**) Transmission image from the region highlighted in red in (A). (**C**) X-ray magnetic circular dichroism (XMCD) images captured when a ± 250 mT magnetic field is applied along the main axis of the ACM. The XMCD contrast lacks the strong black-white contrast seen in Fig. 1c,d, consistent with the near-saturation of magnetization on the tube segments. We attribute the remaining signal to either the misalignment of the applied field with the long axis or to spins which are not fully aligned with the field due to the non-ellipsoidal structure of the tubular screw and its non-uniform demagnetization field.

The laser spot is focused on an ACM and scanned along the long axis of the structure. This allows us to evaluate magnon non-reciprocities by varying the angle between surface normal and the long axis. We attribute the position *z* = 0 μm to the left bulge of the helix as indicated in Fig. 2a. In Fig. 3a–d, spatially resolved Stokes and anti-Stokes spectra taken on a RH ACM and a LH ACM are shown for positive and negative fields. For all BLS spectra, we observe that both the peak frequency and intensity vary characteristically for the Stokes (left) and anti-Stokes (right) spectra as a function of position. The observed asymmetries are reversed by either an opposite field direction or an opposite handedness of the ACM. At *z* = 0 μm on top of the helix the spectra show BLS peaks with local minima of the resonance frequency. At this position, the asymmetric intensity distribution and frequency difference between Stokes and anti-Stokes signals are found to change sign. As reference measurements, we measured the BLS spectrum of a planar nickel thin film prepared in the same batch as the ACMs at 250 mT (Supplementary Fig. 17), where no noticeable non-reciprocity was observed. XMCD data taken on an ACM at 250 mT are shown in Extended Data Fig. 3.

To visualize this asymmetry in the BLS spectra, the resulting Stokes and anti-Stokes line spectra of the RH ACM (pink) and LH ACM (green) are shown at position *z* = −0.7 µm in Fig. 3e. Strikingly, both the intensity and frequency asymmetry between Stokes and anti-Stokes peaks are reversed for the two samples with opposite handedness exposed to the same magnetic field. As the nickel shells were deposited by the same ALD process, and the only difference between the structures is their geometric chirality, we conclude that the sign of the asymmetry parameter *χ* depends on the geometrical handedness of the ACMs. The relative magnitude of frequency non-reciprocity is quantified as the parameter *χ* and evaluated at each scanned position (Fig. 3f). Additionally, the absolute frequency difference Δ*f* has been evaluated (Supplementary Fig. 6). The maximum *χ* amounts to 5.4 × 10^−2^ at *z* = −0.5 μm for the RH ACM. This value for frequency non-reciprocity exceeds the *g*_MChA_ ≈ 1.9 × 10^−2^ that we extracted from ref. ^37^ and which was measured at low temperature for the natural chiral material Cu_2_OSeO_3_.

## Interpreting geometry-induced spontaneous MChA

**Fig. 4:**
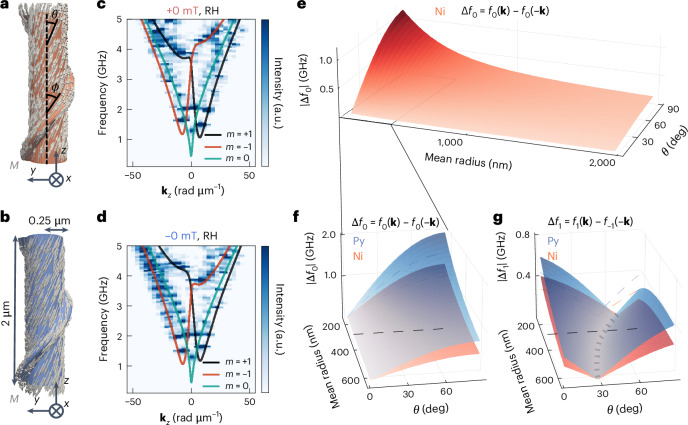
Analysis of non-reciprocity. **a**,**b**, Numerical simulation results of the remanent magnetic textures at +0 mT (**a**) and −0 mT (**b**). The tube’s colour code denotes the sign of the *m*_*z*_ component: red (+) and blue (−). **c**,**d**, Numerical simulation results of the magnon dispersion relations on RH nickel screws at +0 mT (**c**) and −0 mT (**d**). The noisy appearance reflects the finite frequency and spatial resolution of the simulations on ACMs. Analytical calculations of the magnon dispersion of tubes with helical spin texture in the thin-shell limit are shown together with the numerical results. **e**, Optimization of non-reciprocity in terms of frequency asymmetry of the *m* = 0 mode (Δ*f*_0_) by varying geometric and materials parameters and the tilting angle of the helical spins taken with respect to the longitudinal axis. **f**,**g**, Optimization of the Δ*f*_0_ (**f**) and the *m* = ±1 modes (Δ*f*_1_) (**g**) for two different materials: nickel and permalloy (Py).

**Extended Data Fig. 4:**
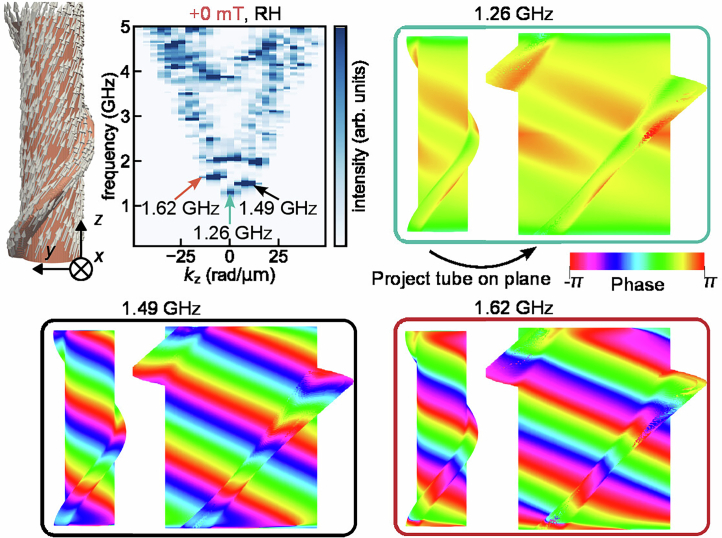
Spatial distribution of the phase of the two band minima. The phase distribution at frequencies matching those of the band minima of the numerically computed dispersion of the RH ACM at +0 mT, shown in Fig. 4b of the main text. The mode profile reveals a ± 2π phase winding for *m* = ±1.

We further analyse and optimize the non-reciprocity by computing the dispersion relation of magnons on the ACM via dynamic micromagnetic simulations and an adapted analytical method (Supplementary Text), respectively. To ensure consistency between the model and numerical results, we focus on magnons propagating along the tubular segment. However, our results indicate that these modes remain coherent throughout the whole structure, including the helix (Extended Data Fig. 4). Figure 4a presents the numerically obtained static helical magnetization for a RH ACM at +0 mT, after initial saturation along the positive *z* direction. The corresponding dispersion is shown in Fig. 4b, where **k**_*z*_ is the wavevector along the tube axis and *m* is the azimuthal mode index which represents the number of 2π phase windings around the perimeter (Supplementary Text, equations (3) and (4)). The colour intensity map shows the eigenfrequencies of the straight tubular segments. The computed band structure reveals a distinct frequency non-reciprocity satisfying $f\left({\mathbf{k}}_{z}\right)\ne f(-{\mathbf{k}}_{z})$, with **k**_*z*_ the wavevector parallel to the tube axis and *f* the frequency. The non-reciprocity at remanence is controlled via the magnetic field history: initially saturating the system with a field applied along the −*z* direction and subsequently reducing this to 0 mT reverses the non-reciprocal dispersion relation of the RH ACM (Fig. 4c,d, −0 mT). The sign of the frequency non-reciprocity is reversed between RH and LH ACMs (Supplementary Fig. 10), substantiating the geometric tunability of directional magnon transport via the surface helix.

We compare the numerically obtained dispersion relation for RH ACMs with analytical calculations for a straight nanotube exhibiting a helical spin texture, following ref. ^28^ and Supplementary Text. Magnons in tubular nanostructures are characterized by the wavevector **k**_*z*_, and the azimuthal mode index *m*. The analytical results (Fig. 4b,d, solid lines) show strong quantitative agreement with the dispersion branches obtained from the numerical simulations. In the analytical model, the bands were computed assuming a straight nanotube with a radius close to half the distance between the simulated helix and the opposing tubular wall. Based on the analytical results, we attribute the frequency minimum near **k**_*z*_ = 0 to the *m* = 0 mode. The two numerically determined band minima near 1.5 GHz correspond to two modes with opposite azimuthal indices *m* = +1 (black) and *m* = −1 (red) (Extended Data Fig. 4). These modes acquire a helical character due to the combined contributions of axial (**k**_*z*_) and azimuthal (*m*) wavevector components. Simulations show that some degree of phase coherence between the tube and helix also remains at higher frequencies (Supplementary Fig. 13).

Non-reciprocity in tubes with helical spin textures arises from two main mechanisms: (1) non-local dipole–dipole interactions, linked to the toroidal moment, and (2) the isotropic exchange interaction, known in this context as the magnon Berry phase, which affects modes with non-zero *m*. The relative strength of both depends on the equilibrium magnetization angle *θ* with respect to the tube axis and the tube radius *r*. In thin tubes, the contributions to the non-reciprocity scale with the tube radius as $\sim \frac{1}{r}$ for dipolar interactions and $\sim \frac{1}{{r}^{2}}$ for exchange interactions. The two aforementioned parameters, *θ* and *r*, are linked to the structural design of the ACM: the angle *θ* depends on the structural helix angle *ϕ* (Fig. 4a), and the tube radius *r* is related to the radius of the ACM’s tubular region. To quantify non-reci procity, we define the frequency difference ${\Delta {f}_{m}}={{f}_{m}}\left({{\mathbf{k}}_{z}}\right)-{{f}_{-m}}(-{{\mathbf{k}}_{z}})$ of the *m* = 0 and *m* = ±1 modes. The frequency difference is **k**_*z*_ dependent (Supplementary Fig. 11), and retains a finite value even in the high |**k**| limit. In the following, we consider a fixed wavevector ${\mathbf{k}}_{z}=\frac{2{{\uppi}}}{\mathrm{pitch}}=3.1\,{\mathrm{rad}}\,{\upmu {\mathrm{m}}}^{-1}$, where pitch = 2 μm is the pitch of the simulated ACM. Figure 4e presents the analytically computed absolute non-reciprocity |Δ*f*_0_| for long tubes as a function of *θ* and mean radius *r*. The non-reciprocity increases with decreasing radius and increasing *θ*, reaching its maximum in the vortex state ($\theta =\frac{\uppi }{2}$), where the toroidal moment is largest. Importantly, pronounced non-reciprocity emerges only at small radii.

To relate the simulated modes with the measured BLS signal, we point out that the μBLS set-up used here has access to wavevectors in the range of **k** = 0–21.3 rad µm^−1^, assuming an optical incident angle ranging from 0° to 90°. The measured signal probably includes a superposition of thermal populations of multiple azimuthal modes, and displays a net non-reciprocity. To compare the magnon modes at 0 mT with those at 250 mT, we analysed the dispersion and frequency non-reciprocity at 0 mT under the above conditions (Supplementary Fig. 11) and at 250 mT, assuming a tilting angle of spins of *θ* = 1° (Supplementary Fig. 12). At 0 mT, both the *m* = 0 and *m* = ±1 modes show a strong **k**-dependent frequency non-reciprocity. In contrast, at 250 mT, the *m* = 0 mode shows a vanishingly small non-reciprocity. This is due to the vanishing of the toroidal moment. The *m* = ±1 modes are split and still show a pronounced frequency non-reciprocity.

Figure 4f shows the non-reciprocity of the *m* = 0 (left) and *m* = ±1 (right) modes for two different ferromagnetic materials: nickel and permalloy. For the *m* = ±1 modes, non-reciprocity reaches its maximum in either the vortex state ($\theta =\frac{\uppi }{2}$) or the axial state (*θ* = 0), depending on the tube radius. Selective excitation of either the *m* = 0 or the *m* = ±1 mode can be achieved by, for example, using an antenna whose dynamic Oersted field efficiently couples to the spatial profile of the desired mode. This selective mode excitation could be advantageous for applications because the *m* = 0 has the highest group velocity near **k**_*z*_ = 0, making it ideal for fast directional signal transport. In contrast, the azimuthal modes display band shifts that enable the excitation of modes with a single sign of phase velocity, while their zero group velocity near **k**_*z*_ = 0 suggests potential diode functionality. Dashed curves in the background of the graph show linecuts taken at *r* = 294 nm, illustrating the dependence of non-reciprocity on the choice of ferromagnetic material (Supplementary Text). It is noteworthy that even under the moderate tilting angle *θ* = 20° considered in Fig. 4a,b, the *m* = 0 already shows a non-reciprocity comparable to the *m* = ±1 modes. The toroidal moment and the associated dipolar contribution scale with the saturation magnetization *M*_s_, whereas the exchange term scales with the ratio of exchange stiffness to saturation magnetization, $\frac{{A}_{\mathrm{ex}}}{{M}_{{\rm{s}}}}$. Our calculations suggest that permalloy exhibits an even stronger non-reciprocity than nickel. Permalloy has been grown previously via ALD and tested in spintronics and magnonics applications^30,38^.

## Conclusions

We report geometrically imprinted spin chirality and have realized spontaneous magnetochiral anisotropy in twisted nickel tubes. By making use of advanced 3D nanostructuring and ALD of nickel, we have overcome traditional dilemmas on chiral magnets which require cryogenic temperatures, applied magnetic fields and complex synthesis. The MChA effect was explored using magnons as information carriers, and a chirality parameter of 5.4 × 10^−2^ was observed, exceeding the corresponding value extracted from magnon non-reciprocity reported for a bulk chiral magnet at cryogenic temperature^37^. Our approach based on inelastic light scattering allowed us to directly resolve the magnon non-reciprocity.

Our work has exploited polycrystalline nickel and hence enables the realization of tunable asymmetric magnon dispersion, independent of the given crystalline structure. Instead, the dispersion asymmetry can be programmed by the pitch or radius of the mesoscale geometry and by the choice of the ferromagnetic material. Such features are especially attractive in hybrid structures with other quasiparticles, for example, phonons, where a separate tuning of non-reciprocal characteristics is desirable. Scaling to smaller radii will become possible via advanced TPL systems with shorter wavelengths or utilizing multiphoton lithography.

Interestingly, magnons in ACMs with a finite azimuthal index have a helical profile. Such magnons are known to have topologically protected finite orbital angular momentum and hence contain an additional degree of freedom, making them compelling for information encoding and microwave signal processing and transmission^39^. MChA should also modify electronic transport^40,41^, thereby producing polarity-dependent resistance. This feature makes the ACMs highly attractive for rectifiers in 3D spintronic applications.

## Methods

### Sample preparation

The magnetic chiral tubes were fabricated by combining TPL and ALD. We applied the additive manufacturing methodology described in ref. ^31^ to 3D polymer wires that contained helical reliefs. These were prepared by TPL using a Photonic Professional GT+ system (Nanoscribe) in three steps. First, negative photoresist IP-Dip was dropped onto a fused-silica substrate (25 × 25 mm^2^, 0.7 mm thick). Second, an infrared femtosecond laser (wavelength, 780 nm; power, 20 mW) was focused inside the resist exploiting the dip-in laser lithography configuration for the exposure. Third, the whole substrate was immersed in propylene glycol monomethyl ether acetate for 20 min and isopropyl alcohol for another 5 min. After the polymer had been dried in ambient conditions, the sample was put into a hot-wall Beneq TFS200 ALD system. We conformally coated the polymer with a 30-nm-thick nickel shell after depositing 5-nm-thick Al_2_O_3_ using the plasma-enhanced ALD process presented in ref. ^28^. The detailed preparation process is presented in Supplementary Fig. 1.

### BLS

The spin dynamics were investigated by µBLS at room temperature (Supplementary Fig. 2). The samples were mounted on a piezo stage, which allowed movement in steps of 50 nm underneath the laser focus. Positive and negative external magnetic fields were applied by permanent magnets mounted in different orientations along the *x* axis, with the ACMs positioned parallel to the *x* axis. A green laser (wavelength, 532 nm) with a power of 3 mW was focused on the surface of the helical magnet using a 100× objective lens with a numerical aperture of 0.75. The full-width at half-maximum of the focused laser spot was experimentally determined to have an upper bound of 436 nm (Supplementary Fig. 15). The s-polarized component of the scattered light was passed through a Glan–Taylor polarizer and directed to a six-pass tandem Fabry–Perot interferometer. In the µBLS set-up, the focused laser light produced a cone of incidence angles around the optical axis of the lens. The backscattered light contained photons that interacted with magnons having different in-plane wavevectors +*k* and –*k*, with *k* magnitudes ranging from 0 to ∼17.7 rad µm^−1^.

### XMCD images

Magnetic chiral tubes of right-handedness (Extended Data Fig. 1a) and left-handedness (Extended Data Fig. 1d) were fabricated on a silicon nitride window membrane. This scaffold supports the ACMs, suspending them over empty space by their ends. These structures were imaged using scanning transmission X-ray microscopy at the UE46_MAXYMUS endstation^42^ of the BESSY II electron storage ring operated by the Helmholtz-Zentrum Berlin für Materialien und Energie. We performed measurements in multibunch hybrid operating mode, where the sample is illuminated by X-rays stroboscopically at a repetition frequency of 500 MHz. We acquired static transmission images using circular polarized monochromatic X-rays with left- and right-handed circularities at the nickel L3 absorption edge (854.5 eV). This energy, slightly offset from the absorption maximum, was chosen to optimize the XMCD signal while minimizing signal loss caused by the thickness of the structures. To remove artificial intensity offsets caused by occasional noise artefacts inherent in the measurement technique (such as the detection of zeroth-order diffracted light, electronic noise from the circuits or thermal fluctuations in the electronics), we applied a dark-field correction to all the transmission images as follows:

$${I}_{\mathrm{corrected}}=\frac{{I}_{\mathrm{sample}}-D}{{I}_{\mathrm{vacuum}}-D}$$

where *D* represents the dark-field factor, which can have values between 0 and 1. For our transmission images, a dark-field factor between 0.9 and 0.92 was applied^43^.

We transformed the transmission images into a dimensionless logarithm scale of normalized intensity, ln(*I*_norm_), using the equation:

$$\mathrm{ln}\left({I}_{\mathrm{norm}}\right)=\mathrm{ln}\left(\frac{{I}_{\mathrm{measured}}}{{I}_{0}}\right)=-\mu t$$

where *I*_measured_ is the intensity of the transmission images measured, *I*_0_ is the reference intensity in the empty space, *µ* is the absorption coefficient (which depends on the circularity of the light) and *t* is the material thickness. To qualitatively determine the relative direction of the magnetization with respect to the X-ray wavevector **k**, we calculated the XMCD factor in each point of the measured transmission images:

$$\mathrm{XMCD}\,\mathrm{factor}\propto {\mu }^{-}-{\mu }^{+}.$$

The resulting XMCD images were processed with a Gaussian filter, using *σ* = 0.5 pixels. This approach gives us estimates of the azimuthal magnetic orientation.

We imaged both RH and LH ACMs using a measurement configuration where the X-rays are incident normally on the structure’s main axis along the $\hat{z}$ direction. This measuring set-up provided sensitivity to the out-of-plane component of the magnetic configuration. The results for the RH ACM (Extended Data Fig. 1a,b), discussed in the main text, reveal that the remanent azimuthal magnetic orientation is determined by the gyration direction of the helix (Extended Data Fig. 1c). A similar behaviour is observed for the LH ACM: the transmission image corresponds to the red-highlighted region in Extended Data Fig. 1d, showing both tubular and helical regions of the ACM (Extended Data Fig. 1e).

XMCD images of the remanent state, measured at *µ*_0_*H* = ±0 mT, show an azimuthally oriented out-of-plane component. As with the RH ACM, this results in a contrast reversal with the direction of the saturating field, confirming that the azimuthal orientation is determined by the helix gyration direction (Extended Data Fig. 1f). When we compare XMCD results for the RH and LH ACMs, we observe that both exhibit similar magnetic patterns but with opposite contrast, indicating that the gyration is reversed between RH and LH ACMs. This implies that the handedness of the magnetic texture is intrinsically determined by the structural chirality of the ACM.

To further understand how the helix direction imprints the gyration direction of the magnetic texture, we present schematics illustrating the X-ray detector view and the projection of the magnetization along the X-ray wavevector view (Supplementary Fig. 3). In the RH ACM, the helix gyration produces a counterclockwise texture for *µ*_0_*H* = +0 mT (Supplementary Fig. 3a) and a clockwise texture for *µ*_0_*H* = −0 mT (Supplementary Fig. 3b). The opposite occurs in the LH ACM, where a clockwise texture is generated with *µ*_0_*H* = +0 mT (Supplementary Fig. 3c) and a counterclockwise texture with *µ*_0_*H* = −0 mT (Supplementary Fig. 3d). Thus, the contrast observed in the XMCD images in Extended Fig. 1 can be explained by the relative projection of the magnetization along the X-ray wavevector, where white contrast appears when the projection is parallel to **k**, and black contrast appears when it is antiparallel.

### Simulation

Micromagnetic simulations were conducted using MuMax^3^ software^44^, which solves the Landau–Lifshitz–Gilbert equation on a finite difference grid. We considered a nickel ACM consisting of a tube with inner radius of 220 nm and a thickness of 30 nm which intersects a hollow helix of ellipsoidal cross-section. The helix had a pitch of 2,000 nm, a diameter of 740 nm, cross-sectional inner major and minor radii of 120 nm and 70 nm, respectively, and a thickness of 30 nm. The helix and tubular segment are directly connected to each other (Supplementary Fig. 5b), and are coupled via both exchange and magnetostatic interactions. The saturation magnetization was set to *M*_s_ = 490 kA m^−1^ and the exchange stiffness to *A*_exc_ = 8 pJ m^−1^ (ref. ^45^). The system was discretized into 160 × 160 × 384 cells of dimension 5 × 5 × 5.2 nm^3^. Six repetitions of periodic boundary conditions along the *z* direction were used.

Hysteresis diagrams of the structures were computed by sweeping an applied field parallel to the tube axis with a 2° misalignment between +1 T and −1 T and back to +1 T. Additionally, a constant background field of 0.7 mT along the *x*,*y* diagonal was applied. The magnetic ground state was computed in between specified field increments by first using the steepest conjugate gradient method^46^ to minimize the energy and then solving the Landau–Lifshitz–Gilbert equation without a precessional term. The resulting ground states provided the initial state for the computation of the toroidal moment and the dynamic behaviour.

The toroidal moment for a given magnetization distribution ${{m}}_{0}({\mathbf{r}})$ was computed per layer according to:

$${\mathbf{\uptau }}\left({{m}}_{0}\right)\mathop{=}\limits^{\mathrm{def}}\frac{1}{A}{\iint }_{A}{\rm{d}}x{\rm{d}}y{\mathbf{r}}\times {{m}}_{0}({\mathbf{r}})$$

with **r** the position vector using the tube axis as the origin and *A* is the area.

The dynamic simulations were conducted as follows. A dynamic field $h={h}_{0}{\mathrm{sinc}}\left(2{{\uppi}}{f}_{{\rm{c}}}\left(t-{t}_{\mathrm{delay}}\right)\right)$ was confined to a strip of width 20 nm along the longitudinal axis of the tube in the centre of the ACM. Here, we used the amplitude *h*_0_ = 3 mT, the cut-off frequency *f*_c_ = 15 GHz and the time offset *t*_delay_ = 26.7 ns. The strip covered only half the cross-sectional area of the ACM to excite both odd- and even-numbered *m* modes. The dynamic field was applied perpendicular to the tube axis. The simulations were run for a total time of 53.3 ns and the magnetization was sampled on the surface of the tube along the tube axis every 33.3 ps. The damping was set to *α* = 10^−3^ and increased quadratically to 1 near the ends of the structure. The dispersion shown in Fig. 4b,d was obtained by performing a 2D fast Fourier transform over the dynamic magnetization sampled on the tube along the *z* axis.

### Analytical dispersion

The simulated dispersion in Fig. 4c,d is plotted together with data obtained from the analytical model proposed by Salazar-Cardona et al.^28^ for nanotubes with helical equilibrium magnetization. The analytical dispersion is given by

$${\omega }_{m}({\mathbf{k}})={\omega }_{M}\left[{{\mathscr{A}}}_{m}({\mathbf{k}})+\sqrt{{{\mathscr{B}}}_{m}(k){C}_{m}({\mathbf{k}})}\right]$$

with $${\omega }_{M}=\gamma {\mu }_{0}{M}_{{\rm{s}}}$$, *γ* is the gyromagnetic ratio and **k** the wavevector. The index *m* denotes the azimuthal mode. $${{\mathscr{A}}}_{m}({\mathbf{k}}),{{\mathscr{B}}}_{m}({\mathbf{k}}),{C}_{m}({\mathbf{k}})$$ are the dynamic stiffness fields. The frequency non-reciprocity is determined by the magnetochiral stiffness field $${{\mathscr{A}}}_{m}({\mathbf{k}})=$$$$-\chi {\mathscr{K}}(m,{\mathbf{k}})\sin \left(\theta \right)+p(N(m,{\mathbf{k}})-\frac{2m{\lambda }_{\mathrm{exc}}^{2}}{{b}^{2}})\cos \left(\theta \right)$$. Here, *θ* is the angle of the magnetization with respect to the tube axis, *b* is the geometrical factor depending on the radius, *λ*_exc_ is the exchange length, *p* = ±1 is the polarity of the magnetization and *χ* = ±1 is the helicity (Supplementary Text). The functions $${\mathscr{K}}(m,{\mathbf{k}}),{\mathscr{N}}\left(m,{\mathbf{k}}_{z}\right)$$ are demagnetizing factors and depend only on the geometry. The analytical data shown in Fig. 4c,d are obtained from equation (18) (Supplementary Text) in the thin-shell approximation where *t* ≈ *λ*_exc_, with *t* the thickness *λ*_exc_. The frequency non-reciprocity sweeps shown in Fig. 4e–g were computed based on equation (18) (Supplementary Text) in the ultrathin-shell approximation where $$t\approx {\lambda }_{\mathrm{exc}}\ll r$$ and *r* is the mean radius of the tube. In all other cases, the dispersion was computed in the thin-shell limit. For the tube sizes under consideration, the two approximations were in good agreement for small values (≲10 rad μm^−1^) of **k**_*z*_. Complete expressions for the dispersion in both approximations are given in Supplementary Text.

The magnetic parameters used for the analytical calculations on nickel are identical to those of the simulations. The thickness of the tube was set to 30 nm. A good quantitative agreement between the analytical theory and the simulations was achieved using an effective mean radius of *r* = 300 nm and a magnetization angle of *θ* = 20° (Supplementary Fig. 8) in the analytical model. Note that this effective radius is larger than the mean radius of the simulated tubular region (235 nm). However, the corresponding mean diameter used for the analytical calculations (600 nm) is almost identical to the cross-sectional mean major-diameter of the ACM (590 nm), that is, the maximum distance between opposing sides along a cross-section of the ACM (Supplementary Fig. 5b). For the computations on permalloy in Fig. 4f,g, we used magnetic parameters *M*_s_ = 800 kA m^−1^ and *A*_exc_ = 13 pJ m^−1^.

## Online content

Any methods, additional references, Nature Portfolio reporting summaries, source data, extended data, supplementary information, acknowledgements, peer review information; details of author contributions and competing interests; and statements of data and code availability are available at 10.1038/s41565-025-02055-3.

## Supplementary information

Supplementary Information Supplementary Text and Figs. 1–18.

## Data Availability

All the data presented in this work are available via Zenodo at 10.5281/zenodo.17223765 (ref. ^47^).

## Code availability

The code used for the micromagnetic simulations presented in this work is available via Zenodo at 10.5281/zenodo.17223765 (ref. ^47^).
