# Supplementary material for: Geometry-induced spin chirality in a non-chiral ferromagnet at zero field
Source: Nat Nanotechnol. 2025 Dec 4;21(1):58–64. doi: 10.1038/s41565-025-02055-3 (PMC12819137; doi:10.1038/s41565-025-02055-3)
Supplement: Supplementary file 1 — Supplementary Text and Figs. 1–18. [file 41565_2025_2055_MOESM1_ESM.pdf]

nature nanotechnology

Supplementary information https://doi.org/10.1038/s41565-025-02055-3

Geometry-induced spin chirality in a non-chiral ferromagnet at zero field

In the format provided by the authors and unedited

Table of contents:

Supplementary Text

Supplementary Figs. S1-S18

SUPPLEMENTARY TEXT

Chiral Parameter χ

To quantitatively analyse the asymmetries in frequency as a function of spot position (Fig.3A-D), we apply a Lorentzian fit to the peak of each Stokes and anti-Stokes spectrum according to

$$I_{\text{total}} = I_0 + \frac{I}{(f - f_{\text{c}})^2/\Gamma_f^2 + 1}, \tag{S1}$$

where Γ_f is the half width at half maximum (HWHM), f_{c} is the central frequency, I is the signal strength at the maximum, and I_0 is the background signal in the spectrum. We then calculate $\Delta f = |f_{\text{c,+k}}| - |f_{c,-k}|$ and $\Delta I = I_{+k} - I_{-k}$ with $+k, -k$ defined as in Fig. 3. However, a systematic frequency shift f', independent of z, is inevitably present due to a slight misalignment of the laser scanning path with respect to the center of the magnetic tubes. In order to compensate for this systematic offset, we introduce $\Delta f' = \Delta f - f'$ and $\Delta I' = \Delta I - I'$, representing the position-dependent component of Δf and ΔI. Using the extracted parameters $\Delta f'$ and $\Delta I'$, we calculate the asymmetry parameters as a function of z-position. The parameters $\Delta I'$ for RH- and LH-ACMs at $\pm$ 250 mT (red and blue symbols) are summarized in Fig. S4 The parameters Δf and ΔI for RH- and LH-ACMs at $\pm$ 250 mT are summarized in Fig. S6.

To analyze the relative strength of the magnetochiral effect in ACMs and compare them to natural materials, we evaluate the relative magnitude of frequency nonreciprocity according to

$$\chi(\mathbf{H}) = \frac{\Delta f'(+\mathbf{H})}{f_{+k}(+\mathbf{H}) + f_{-k}(+\mathbf{H})} - \frac{\Delta f'(-\mathbf{H})}{f_{+k}(-\mathbf{H}) + f_{-k}(-\mathbf{H})} \tag{S2}$$

where f_{+k} refers to $|f_c, +k|$ and f_{-k} refers to $|f_c, -k|$. We note that the chiral parameter χ is nominally equivalent to g_{MCh} as defined in Refs. (*1, 2*). In Fig. 3f we display χ as a function of position for the RH-ACM (pink) and the LH-ACM (green). Each dataset is anti-symmetric with respect to $z = 0$. The consistent anti-phase variations observed for all three different asymmetry parameters as a function of BLS spot position are a clear signature of chirality effects controlled by geometrical handedness and magnetic field direction. The maximum peak-to-peak difference amounts to 5.4×10^{-2} at $z = -0.5\ \mu$m for RH-ACM. This value is more than three orders of magnitude larger than g_{MCh} reported for a natural chiral material ($g_{\text{MCh}} \approx 40 \times 10^{-6}$) in Ref. (*1, 2*).

Simulations on a 4000 nm long ACM

Simulations were performed on a left-handed 4000 nm long ACM with pitch 2000 nm, tube outer diameter 125 nm, helix outer diameter 200 nm, and thickness 30 nm, following similar methodology as described in the Methods. The damping was quadratically increased to 1 over 800 nm at both ends. The resulting equilibrium magnetization after saturating the structure along the positive z-direction is shown in Fig. S7a (d) for the case without (with) periodic boundaries. In Fig. S7a, the stray field induced curling of the magnetization due to its finite size is clearly seen at the end of the tubes whereas the magnetization in the center of the structure follows the helix. Spin waves were exciting using a signal described in the Methods which covered the entire cross sectional area. The resulting dispersion at ±0 mT states is shown in Fig. S7b,c. Here, to compute the dispersion only the data from the middle of the tube was used (Fig. S7a lower panel). The dispersion of the same structure with periodic boundaries and hence with the magnetization in the helical state throughout the tube (Fig. S7d,e,f) is qualitatively very similar to that shown in Fig. S7b,c, verifying the validity of the results with periodic boundaries shown in Fig. 4.

Analytical dispersion

Here we provide the full equations and parameters used for the analytical results of Fig. 4. The equations are based on the model provided by Salazar-Cardona et al. for the magnon dynamics in thin nanotubes with helical spin textures (28) of the main text.

Consider an infinitely long ferromagnetic nanotube aligned along the z-axis with saturation magnetization M_s, exchange stiffness A_exc, gyromagnetic ratio γ, and inner and outer radii r_i and r_o respectively, mean radius, thickness and cross-sectional area $r = (r_\text{i} + r_\text{o})/2$, $T = r_\text{o} - r_\text{i}$, and $S = 2\pi r T$. The exchange length is $\lambda_\text{exc} = \sqrt{\frac{A_\text{exc}}{\frac{1}{2}\mu_0 M_\text{s}^2}}$. For a given equilibrium magnetization distribution $\mathbf{m}_0$, the dynamical magnetization components are $\mathbf{m} = m_\rho \hat{\rho} + m_v \hat{v}$. Here, $\hat{\rho}$ is the radial coordinate and $\hat{v} = \hat{\rho} \times \mathbf{m}_0$ is a coordinate locally orthogonal to both $\hat{\rho}$ and $\mathbf{m}_0$. These dynamic components can be expanded as:

$$m_\rho(\varphi, z, t) = \sum_{m=-\infty}^{\infty} \int_{-\infty}^{\infty} dk_z e^{ik_z z} e^{im\varphi} e^{-i\omega_m(k_z)t} \mathcal{R}_{k_z}^m \tag{S3}$$

$$m_v(\varphi, z, t) = \sum_{m=-\infty}^{\infty} \int_{-\infty}^{\infty} dk_z e^{ik_z z} e^{im\varphi} e^{-i\omega_m(k_z)t} \mathcal{V}_{k_z}^m. \tag{S4}$$

Here, φ is the azimuthal coordinate, k_z is the wavevector along the tube axis, m is the azimuthal mode index, ω_m is the frequency corresponding to mode m, and $\mathcal{R}_{k_z}^m, \mathcal{V}_{k_z}^m$ are amplitude projections of oscillations along the $\hat{\rho}$ and $\hat{v}$ directions, respectively.

The self-demagnetization fields for thin-shells ($T \approx \lambda_{\text{exc}}$) are computed via the following hyper geometrical functions (25):

$$\mathcal{J}(m, k_z) = \frac{\pi}{S} \int_0^{\infty} dq \frac{q^3}{2\left(q^2 + k_z^2\right)} \left[\Gamma_m(q)\right]^2 \tag{S5}$$

$$\mathcal{K}(m, k_z) = \frac{\pi}{S} \int_0^{\infty} dq \frac{q^2 k_z}{q^2 + k_z^2} \Gamma_m(q) \Lambda_m(q) \tag{S6}$$

$$\mathcal{L}(m, k_z) = \frac{\pi}{S} \int_0^{\infty} dq \frac{2qk_z^2}{q^2 + k_z^2} \left[\Lambda_m(q)\right]^2 \tag{S7}$$

$$\mathcal{M}(m, k_z) = m\frac{\pi}{S} \int_0^{\infty} dq \frac{2qk_z}{q^2 + k_z^2} \Lambda_m(q) \mathcal{I}_m(q) \tag{S8}$$

$$\mathcal{N}(m, k_z) = m\frac{\pi}{S} \int_0^{\infty} dq \frac{q^2}{q^2 + k_z^2} \Gamma_m(q) \mathcal{I}_m(q) \tag{S9}$$

$$\mathcal{O}(m, k_z) = m^2\frac{\pi}{S} \int_0^{\infty} dq \frac{2q}{q^2 + k_z^2} \left[\mathcal{I}_m(q)\right]^2 \tag{S10}$$

where $\mathcal{I}_m(q) = \int_{r_i}^{r_0} d\rho J_m(q\rho)$, $\Lambda_m(q) = \int_{r_i}^{r_o} d\rho\ \rho J_m(q\rho)$, and $\Gamma_m(q) = \Lambda_{m-1}(q) - \Lambda_{m+1}(q)$, and $J_m(x)$ is the Bessel function of the first kind and order m. These factors depend only on geometrical parameters of the tube, the wavevector k_z, and azimuthal mode number m. For a given set of tube dimensions, these functions are solved for by numerical integration.

To get around the need for this time-consuming numerical integration, the functions can be simplified assuming the ultra-thin limit where $T \approx \lambda_{\text{exc}} \ll r$. In this case, the factors can be written in terms of the modified Bessel functions I_m and K_m:

$$\begin{aligned}
\mathcal{J}(m,k_z) &\approx 1 + rT\frac{\partial I_m(|k_z|r)}{\partial r}\frac{\partial K_m(|k_z|r)}{\partial r} && \text{(S11)}\\
\mathcal{K}(m,k_z) &\approx \frac{k_z RT}{2}\frac{\partial}{\partial r}I_m(|k_z|r)K_m(|k_z|r) && \text{(S12)}\\
\mathcal{L}(m,k_z) &\approx RTk_z^2 I_m(|k_z|r)K_m(|k_z|r) && \text{(S13)}\\
\mathcal{M}(m,k_z) &\approx mk_zTI_m(|k_z|r)K_m(|k_z|r) && \text{(S14)}\\
\mathcal{N}(m,k_z) &\approx \frac{mT}{2}\frac{\partial}{\partial r}I_m(|k_z|r)K_m(|k_z|r) && \text{(S15)}\\
\mathcal{O}(m,k_z) &\approx m^2\frac{T}{r}I_m(|k_z|r)K_m(|k_z|r)\,. && \text{(S16)}
\end{aligned}$$

In the helical state, the orientation of the dimensionless equilibrium magnetization $\mathbf{m}_0$ can be specified by the angle θ between the magnetization and the z-axis. If θ is limited to the interval $[0, \pi/2]$ with $\theta = 0$ corresponding to the axial state and $\theta = \pi/2$ corresponding to the vortex state, the magnetization can be decomposed as:

$$\mathbf{m}_0 = \chi \sin(\theta)\,\hat{e}_\varphi + p\cos(\theta)\,\hat{e}_z, \tag{S17}$$

with $\chi, p \in \{-1, +1\}$ the helicity and polarization respectively and $\hat{e}_\varphi$ and $\hat{e}_z$ the azimuthal and axial unit vectors.

The fields $\mathcal{A}, \mathcal{B}, \mathcal{C}$ that show up in the dispersion for spin waves in the helical state:

$$\omega_m(k_z) = \omega_M\left[\mathcal{A}_m(k_z) + \sqrt{\mathcal{B}_m(k_z)\,\mathcal{C}_m(k_z)}\right] \tag{S18}$$

can be expressed, assuming the absence of uniaxial anisotropy and applied field, as

$$\begin{aligned}
\mathcal{A}_m(k_z) &= -\chi\mathcal{K}(m,k_z)\sin(\theta) + p\left[\mathcal{N}(m,k_z) - \frac{2m\lambda_{\text{exc}}^2}{b^2}\right]\cos(\theta) && \text{(S19)}\\
\mathcal{B}_m(k_z) &= \lambda_{\text{exc}}^2\left(k_z^2 + \frac{m^2}{b^2}\right) + \frac{\lambda_{\text{exc}}^2}{b^2}\cos^2(\theta) + \mathcal{J}(m,k_z) && \text{(S20)}\\
\mathcal{C}_m(k_z) &= \lambda_{\text{exc}}^2\left(k_z^2 + \frac{m^2}{b^2}\right) + \frac{\lambda_{\text{exc}}^2}{b^2}\cos(2\theta) + \mathcal{O}(m,k_z)\cos^2(\theta) && \text{(S21)}\\
&\quad -\chi p\mathcal{M}(m,k_z)\sin(2\theta) + \mathcal{L}(m,k_z)\sin^2(m,k_z)
\end{aligned}$$

where we have introduced $\frac{1}{b^2} = \frac{1}{rT}\ln\left(\frac{r+T/2}{r-T/2}\right)$.

Magnon version of Knife-edge experiment

To determine the laser spot diameter, a magnon version of knife-edge experiment is performed using the BLS system. As shown in Fig. S15a , the laser spot scans across a silicon nitride frame coated with a 30 nm Ni thin film via ALD, under a 250 mT magnetic field. The magnon signal gradually disappears as the laser crosses the edge.

We assume intensity profile of the knife-edge experiment follows a Gaussian cumulative distribution function (CDF). The fitting function, incorporating a negative sign before the error function, is given by:

$$P(x) = \frac{P_0}{2}\left[1 - \mathrm{erf}\left(\frac{x - x_0}{\sqrt{2}\sigma}\right)\right] \tag{S22}$$

where $P(x)$ is the intensity as a function of position x, P_0 is the maximum intensity, x_0 is the position where the intensity reaches half of P_0 (transition midpoint), and σ is the standard deviation of the Gaussian beam.

The error function is defined as:

$$\mathrm{erf}(z) = \frac{2}{\sqrt{\pi}} \int_0^z e^{-t^2} dt \tag{S23}$$

This function characterizes the gradual intensity variation observed as a knife edge traverses a Gaussian beam. The BLS intensity was integrated over the frequency range from -17 to -10 GHz. By fitting the measured intensity profile to the cumulative distribution function (CDF) of a Gaussian, the extracted beam width was determined to be $\sigma = 0.185\ \mu$m, corresponding to a full width at half maximum (FWHM) of $2\sqrt{2\ln 2} \cdot \sigma \approx 2.355 \cdot \sigma = 436$ nm. This experimentally obtained FWHM is close to the theoretical diffraction-limited value for a Gaussian beam, given by $0.51 \cdot \frac{\lambda}{\mathrm{NA}} = 362$ nm, based on a laser wavelength of $\lambda = 532$ nm and a numerical aperture of NA $= 0.75$. It should be noted that inhomogeneities in the magnetic properties near the sample edge may also contribute to the attenuation of the magnon signal; therefore, this magnon-based knife-edge measurement reflects the upper limit of the actual laser spot size.

SUPPLEMENTARY FIGURES

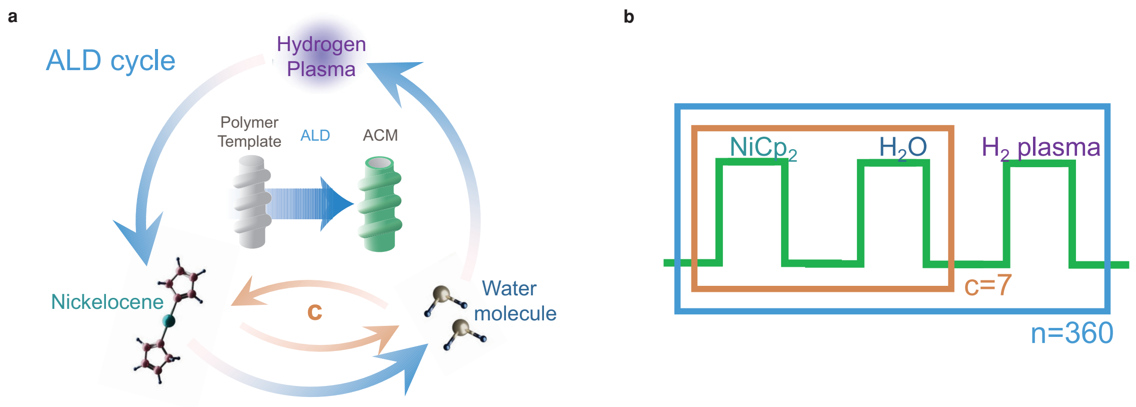

Figure S1: **Plasma-Enhanced Atomic Layer deposition (ALD)**. (**A**) Sketch of an ALD cycle for Ni. The ALD cycle consists of c steps of $NiCp_2/H_2O$ (orange arrows), resulting in the formation of nickel oxide, and followed by a hydrogen plasma (purple), reducing nickel oxide to metallic nickel. This cycle was repeated n times as in (**B**). 30 nm conformal Ni coating was achieve by $n = 360$ while keeping $c = 7$ at a substrate temperature of $T = 180°C$. Then an in-situ annealing process at $350°C$ under a mixture of pure hydrogen and nitrogen flow was performed.

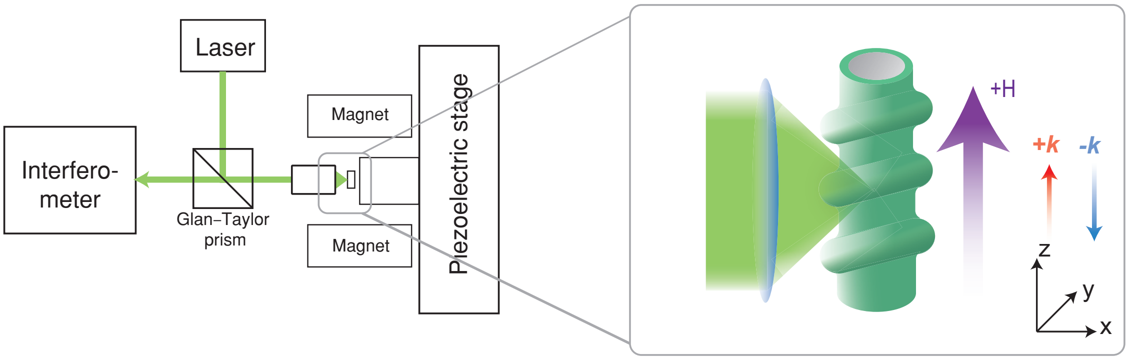

Figure S2: **Illustration of a room temperature μ-BLS**. The ACMs are suspended from the substrate and mounted to a piezostage. S-polarized green laser light is selected using a Glan-Taylor prism and then focused by a 100x objective onto the samples. The inelastically scattered light is collected by the same objective, and its p-polarized components are subsequently sent to interferometer.

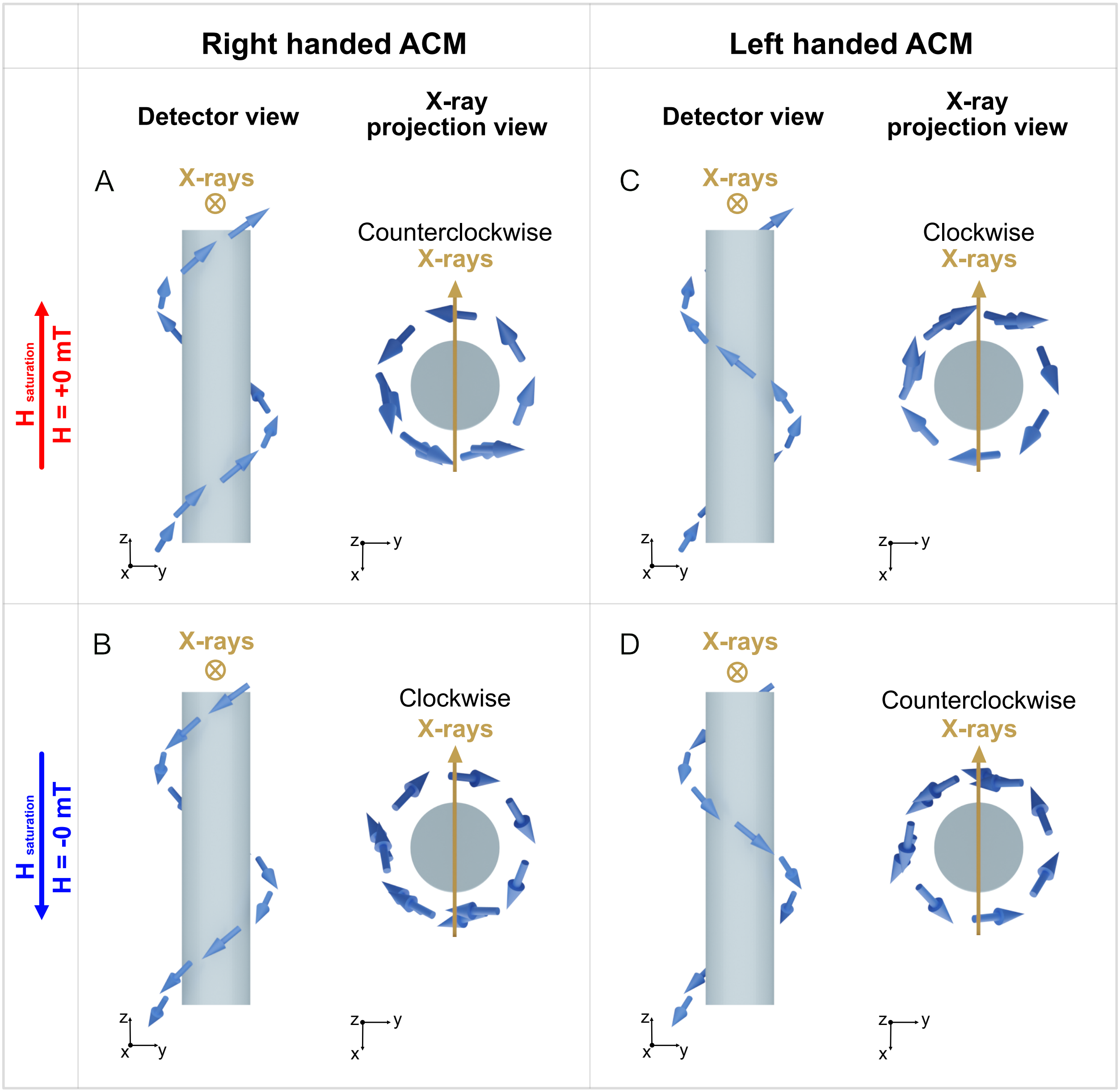

Figure S3: **Schematics of the screw-like textures for RH and LH ACMs, showing detector and X-ray wave vector projection views.** **(A, B)** RH ACM in remanence at $\mu_0 H = \pm 0$ mT after applying a saturating field of (±250 mT). The magnetization projection along $\vec{k}$ reveals a counterclockwise texture for positive saturation **(A)** and a clockwise texture for negative saturation **(B)**. **(C, D)** LH ACM under the same conditions, but with reversed textures: clockwise for positive saturation **(C)** and counterclockwise for negative saturation **(D)**.

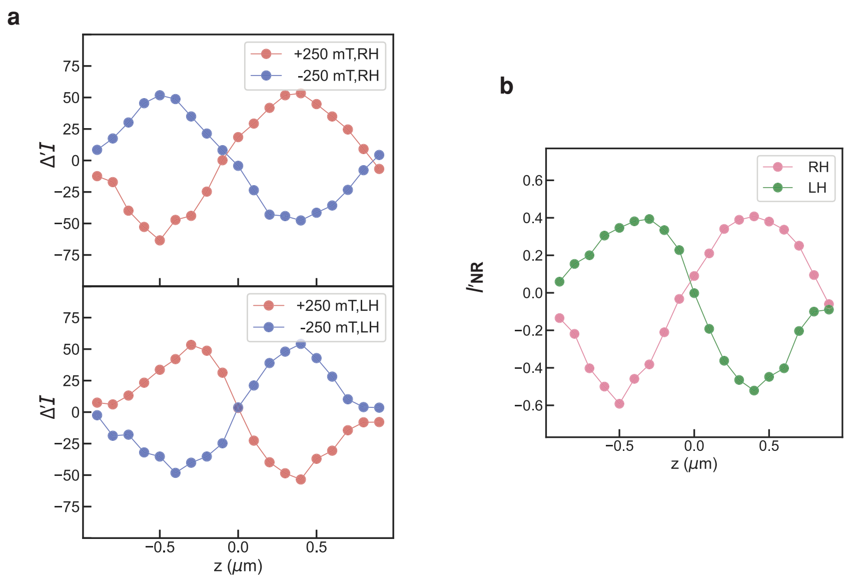

Figure S4: **Intensity nonreciprocity.** (**A**) Quantitative analysis of the nonreciprocity in terms of the magnitude difference of $\Delta I'$ for the RH-ACM (top) and the LH-ACM (bottom). (**B**) The magnitude of $I'_{\mathrm{NR}}(\mathbf{H})$ for the RH-ACM (pink) and LH-ACM (green) as a function of position z along the tube (Fig. S2).

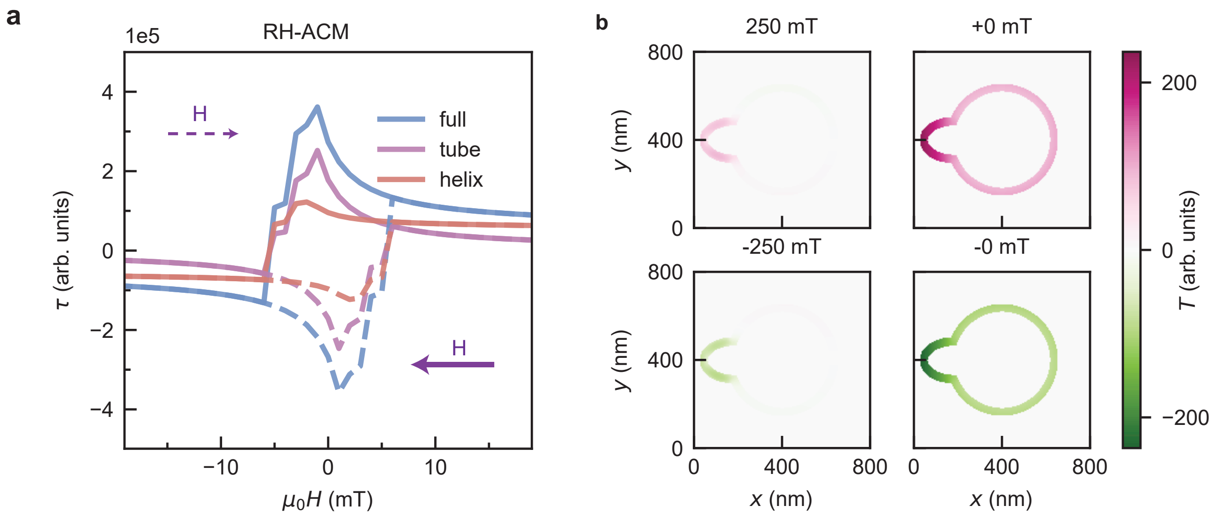

Figure S5: **Field dependence of the toroidal moment of a right handed ACM.** (**A**) Toroidal moment τ of a RH-ACM (blue curve) simulated as a function of axial field H and its sweep direction indicated by arrows. The magenta and orange curves decompose the toroidal moment of the RH-ACM in the contributions from the tube and helix regions, respectively. The field was swept from negative to positive (dashed lines) and back (solid lines). The hysteretic behavior enables a reprogrammable $\boldsymbol{\tau}$ at $H = 0$. **B** Simulated cross-section of τ distribution on a RH-ACM when varying the magnetic field from +250 mT, to +0 mT, −250 mT and −0 mT. The cross-section was taken at the central plane of the ACM.

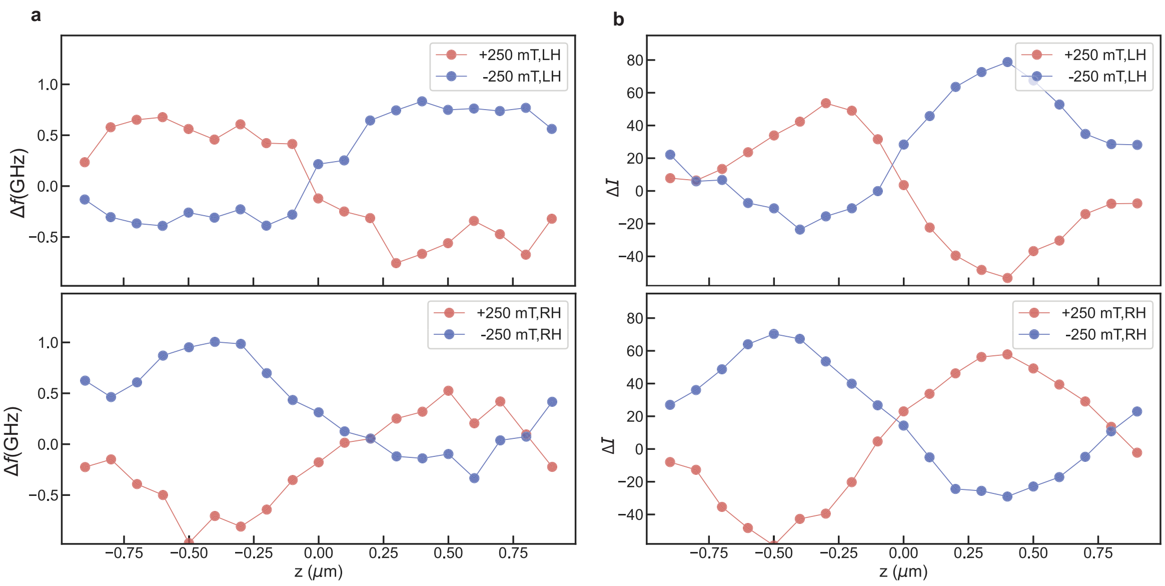

Figure S6: **Frequency difference** Δf **and intensity difference** ΔI**.** (**A**) Quantitative analysis of the nonreciprocity in terms of the frequency difference Δf for the LH-ACM (top) and the RH-ACM (bottom) as a function of position z. (**B**) Similar analysis as in (A) for the intensity difference ΔI.

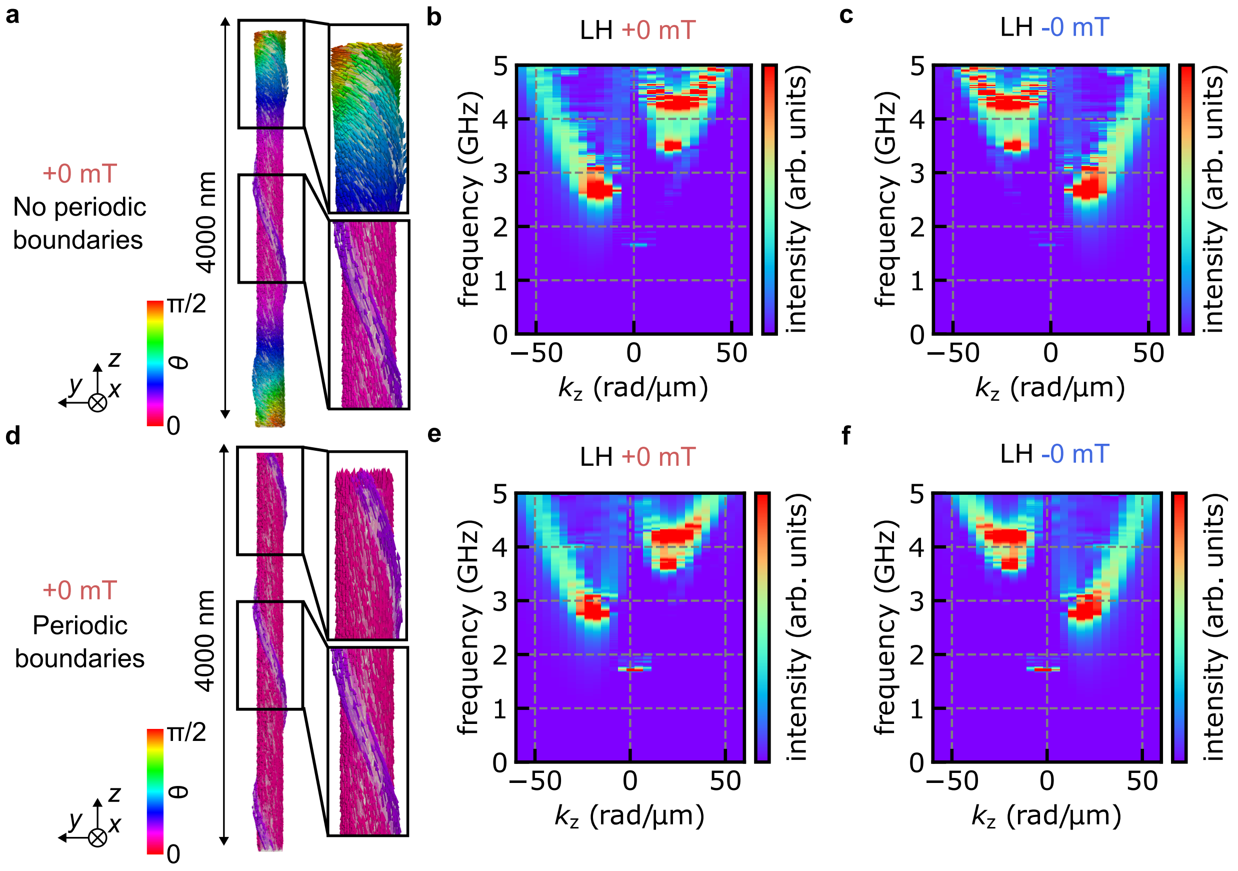

Figure S7: **Dispersion computed for a 4000 nm long ACM.** (**A**) Remanent state after saturating along the positive z-direction using periodic boundaries. (**B**), (**C**) Dispersion for the 4000 nm long ACM with periodic boundaries for remanent states +0 mT and -0 mT, respectively. (**D**) Remanent state after saturating along the positive z-direction without periodic boundaries. (**E**), (**F**) Dispersion for the 4000 nm long ACM without periodic boundaries for remanent states +0 mT and -0 mT, respectively.

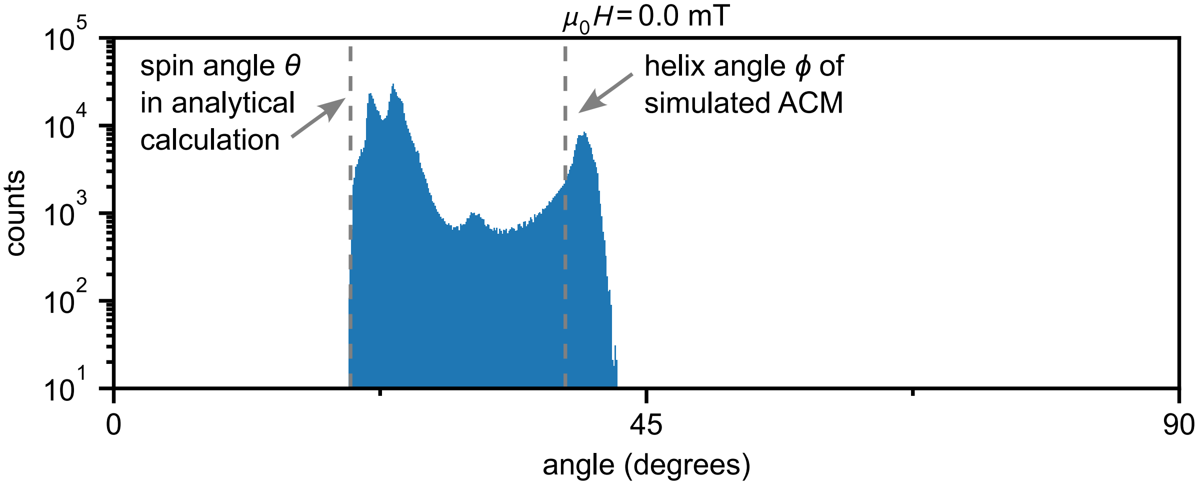

Figure S8: **Numerically computed distribution of the magnetization angle with respect to the longitudinal axis of an ACM at +0 mT.** The left vertical dashed lines indicates an angle of 20° which was used as the spin angle θ for the analytical dispersion shown in Fig. 4b,d of the main text. The right vertical dashed line corresponds to the structural helix angle ϕ = 38° of the simulated ACM. A spin angle θ slightly lower than the main peaks in the distribution was chosen for the analytical calculations, as this yielded better correspondence with the numerically computed dispersion.

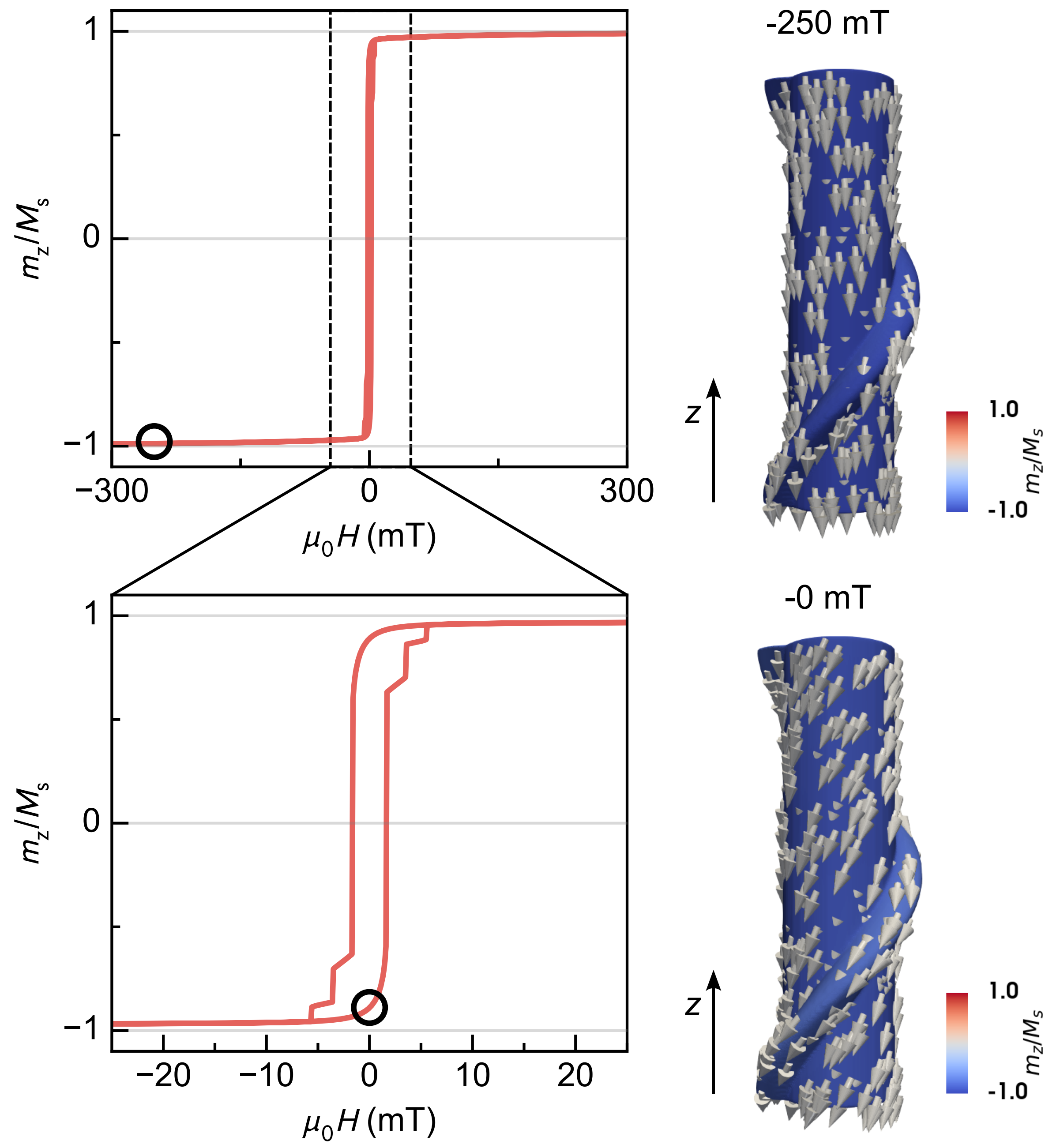

Figure S9: **Numerically computed hysteresis and magnetization textures at -250 mT and -0 mT.** The circles mark the fields at which the magnetization is visualized on the right. The arrows indicate the local spin orientation and the color coding corresponds to the normalized z-component of the magnetization m_z. The applied field H is along the z-direction, as indicated by the arrow.

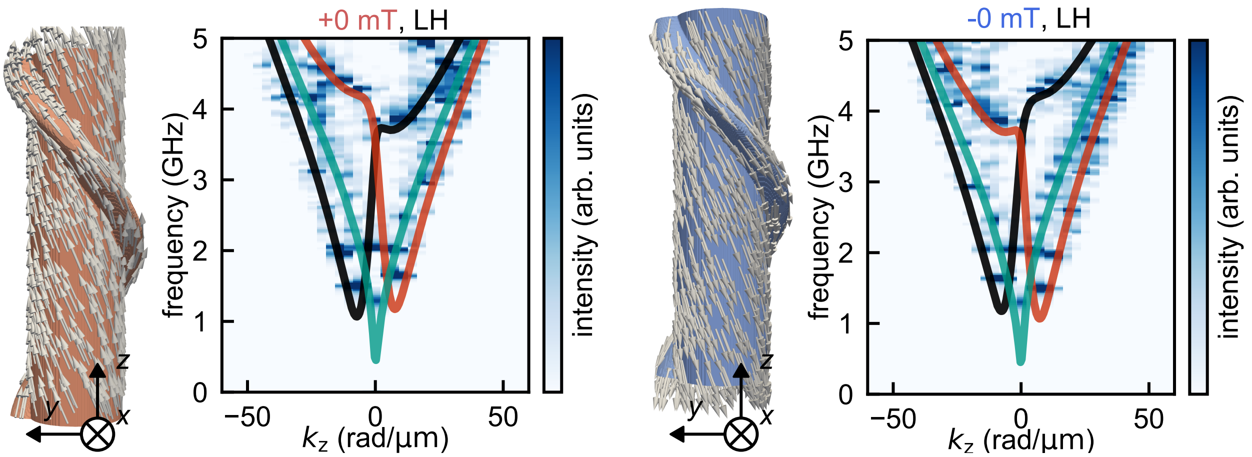

Figure S10: **Numerically computed magnetization distribution and dispersion of a LH ACM at ±0 mT.** Structural and magnetic parameters are identical to the RH ACM shown in Fig. 4a-d of the main text.

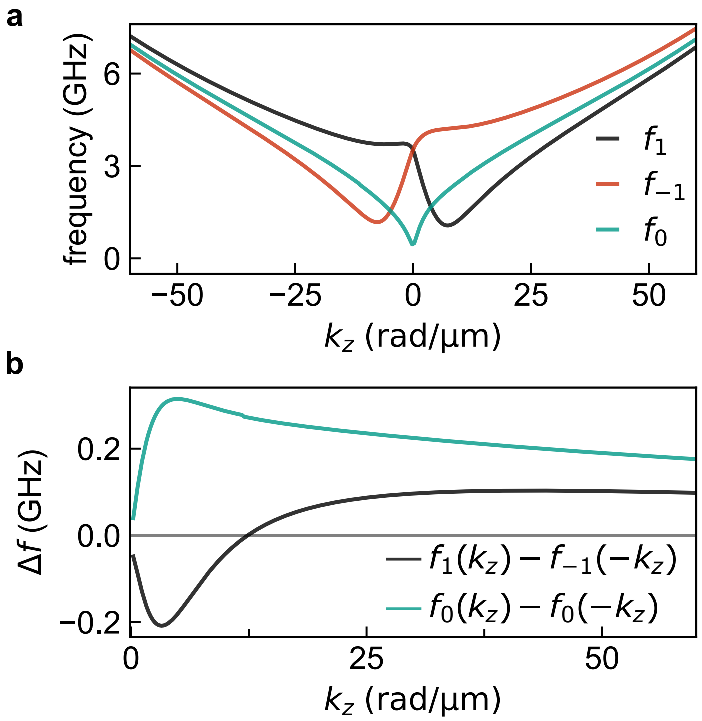

Figure S11: **Analytical dispersion and frequency non-reciprocity at 0 mT** (**A**) Dispersion corresponding to the RH +0 mT state of Fig. 4a,b of the main text over a wider range of wavevectors. (**B**) Frequency nonreciprocity Δf computed for the $m = \pm 1$ (green) and $m = 0$ (black) modes

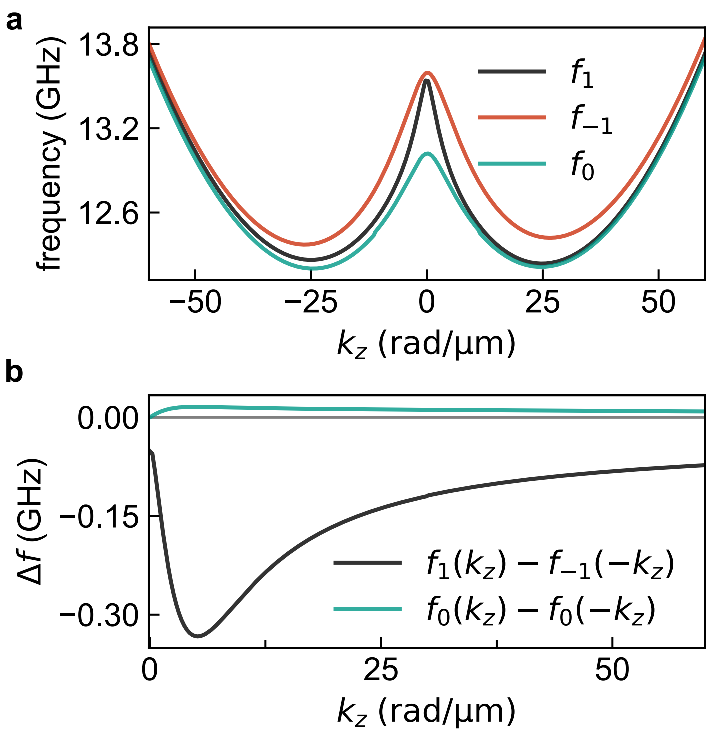

Figure S12: **Analytical dispersion and frequency non-reciprocity at 250 mT** (**A**) Dispersion corresponding to the RH +250 mT state. (**B**) Frequency nonreciprocity Δf computed for the $m = \pm 1$ (green) and $m = 0$ (black) modes

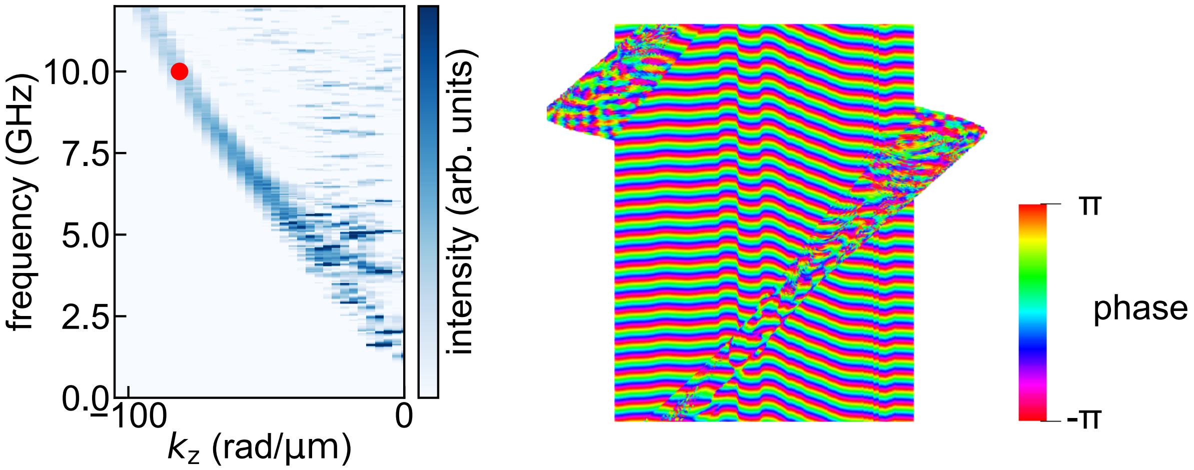

Figure S13: **Phase of a mode at 10 GHz.** Numerically simulated dispersion at higher frequencies than in the main text (left), and extracted phase distribution of the dynamic magnetization at $f = 10$ GHz and $k = -81.47$ rad/μm (right). The phase was extracted via a 2D FFT of the dynamic magnetization, filtering out the $k_z = -81.47$ rad/μm component, and performing a 1D spatial inverse FFT.

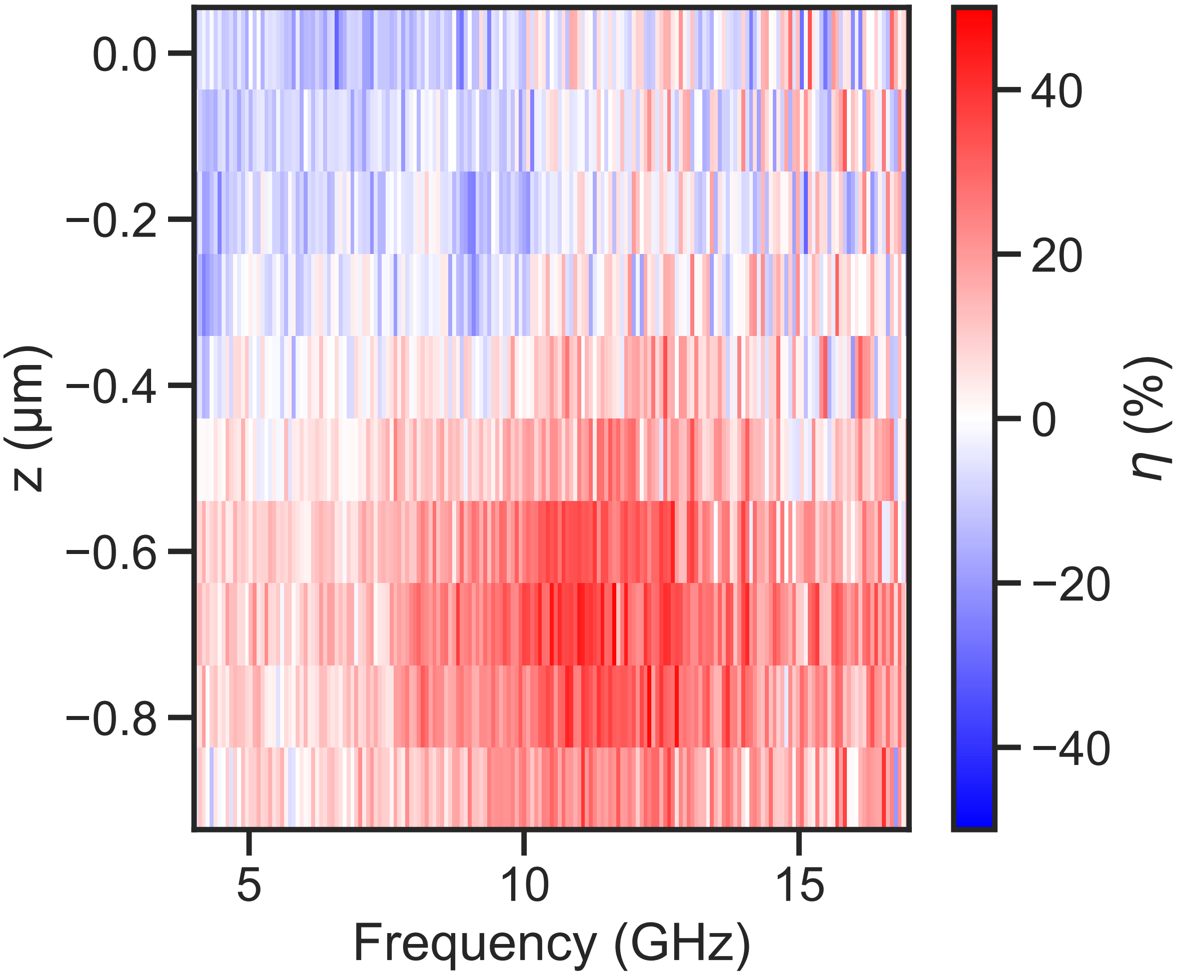

Figure S14: **Frequency dependence of the nonreciprocity.** η (%) as a function of frequency (GHz) and position $z(\mu m)$ on a RH-ACM at +0 mT. The colorscale represent variation of η, with red indicating positive values and blue indicating negative values.

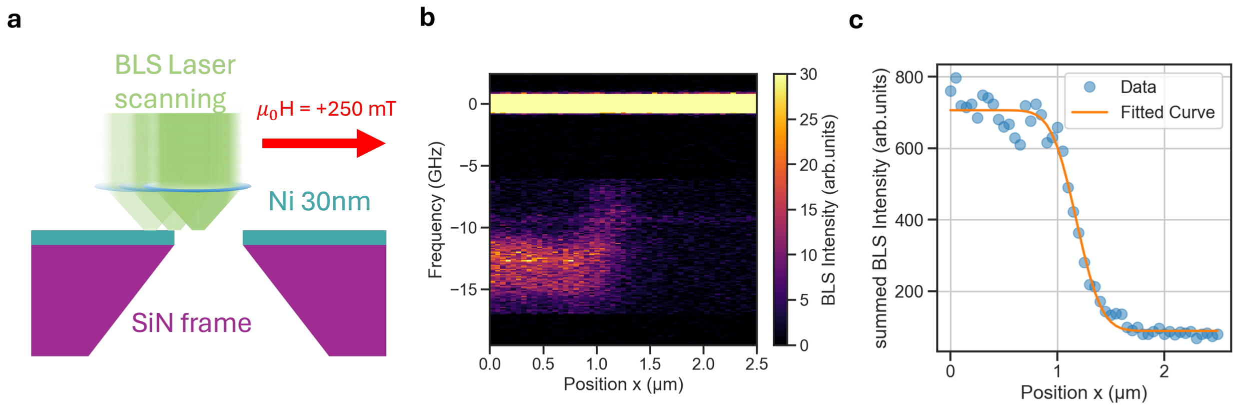

Figure S15: **Magnon Knife-edge experiment. (A)** Schematic of the experiment setup. **(B)** Brillouin light scattering spectra of magnons on Ni thin film depending on the position x. The scanning step size is 0.05 μm. **(C)** Position dependence of the summed intensity for the frequency range of -10 to -17 GHz, with the knife-edge fitting curve overlaid. The curve represents the fitted intensity profile used to determine the laser spot size. The fitting result yields a standard deviation of the Gaussian beam profile $\sigma = 0.185$ μm, corresponding to a laser spot size of approximately 0.436 μm.

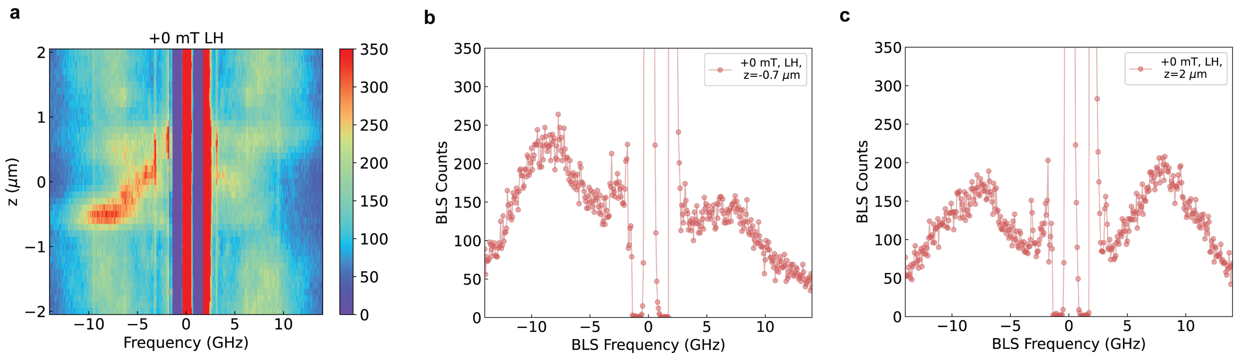

Figure S16: **BLS spectra of LH-ACMs at remanence (+0 mT). (A)** BLS spectra measured as a function of position across the spiral (see definition of z in Fig. 2a) along an LH-ACM in a field of a +0 mT. **(B,C)** Local magnon spectra at $z = -0.7$ μm (helix segment) and $z = 2$ μm (tube segment), respectively, measured in the remanent state (+0 mT) after applying a +30 mT field.

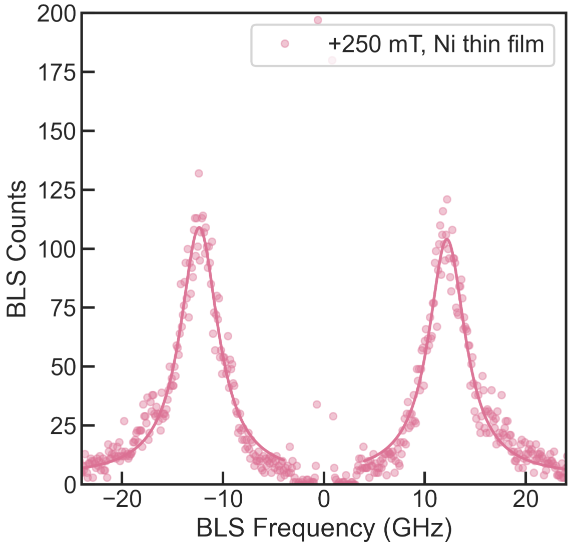

Figure S17: **BLS spectrum on plane film Ni at 250 mT.** Magnon spectra measured on a Ni thin film sample (red symbols) under an applied field of +250 mT. The curves represent Lorentzian fits, which yield a frequency difference of $\Delta f = 0.153$ GHz. This value is comparable to the frequency resolution of the BLS setup, defined by a frequency step of 0.1 GHz.).

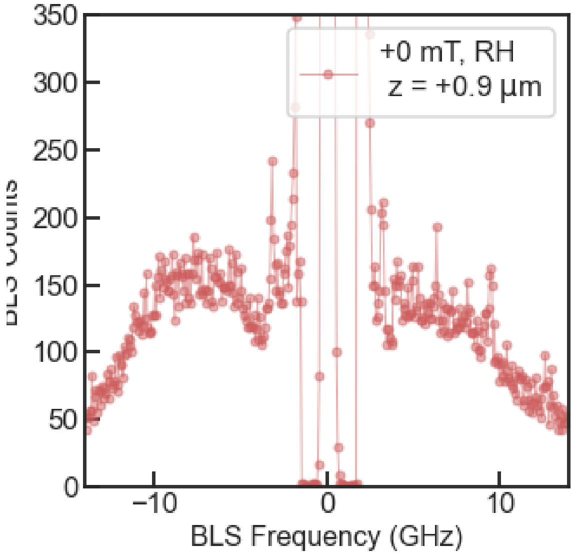

Figure S18: **BLS spectra of RH-ACMs at +0 mT at z = +0.9** μm**.** Local magnon spectra at z = +0.9 μm measured in the remanent state (+0 mT) after applying a +30 mT field on a RH-ACMs.

References and Notes

1. T. Nomura, *et al.*, Phonon magnetochiral effect. *Physical Review Letters* **122** (14), 145901 (2019), https://doi.org/10.1103/PhysRevLett.122.145901.

2. T. Nomura, *et al.*, Nonreciprocal Phonon Propagation in a Metallic Chiral Magnet. *Physical Review Letters* **130** (17), 176301 (2023), doi:10.1103/PhysRevLett.130.176301, https://doi.org/10.1103/PhysRevLett.130.176301.
